# Advances on Axial Coordination Design of Single-Atom Catalysts for Energy Electrocatalysis: A Review

**DOI:** 10.1007/s40820-023-01196-1

**Published:** 2023-10-13

**Authors:** Linjie Zhang, Na Jin, Yibing Yang, Xiao-Yong Miao, Hua Wang, Jun Luo, Lili Han

**Affiliations:** 1 State Key Laboratory of Structural Chemistry, Fujian Institute of Research on the Structure of Matter, Chinese Academy of Sciences, Fuzhou, 350002 People’s Republic of China; 2 College of Chemistry and Materials Science, Fujian Normal University, Fuzhou, 350117 People’s Republic of China; 3 State Key Laboratory of ASIC and System, Shanghai Institute of Intelligent Electronics and Systems, School of Microelectronics, Fudan University, Shanghai, 200433 People’s Republic of China; 4 ShenSi Lab, Shenzhen Institute for Advanced Study, University of Electronic Science and Technology of China, Shenzhen, 518110 People’s Republic of China

**Keywords:** Single-atom catalyst, Axial coordination, Synthetic strategy, Electrocatalytic application

## Abstract

Single-atom catalysts (SACs) have garnered increasingly growing attention in renewable energy scenarios, especially in electrocatalysis due to their unique high efficiency of atom utilization and flexible electronic structure adjustability. The intensive efforts towards the rational design and synthesis of SACs with versatile local configurations have significantly accelerated the development of efficient and sustainable electrocatalysts for a wide range of electrochemical applications. As an emergent coordination avenue, intentionally breaking the planar symmetry of SACs by adding ligands in the axial direction of metal single atoms offers a novel approach for the tuning of both geometric and electronic structures, thereby enhancing electrocatalytic performance at active sites. In this review, we briefly outline the burgeoning research topic of axially coordinated SACs and provide a comprehensive summary of the recent advances in their synthetic strategies and electrocatalytic applications. Besides, the challenges and outlooks in this research field have also been emphasized. The present review provides an in-depth and comprehensive understanding of the axial coordination design of SACs, which could bring new perspectives and solutions for fine regulation of the electronic structures of SACs catering to high-performing energy electrocatalysis.

## Highlights

- The burgeoning research topic of axially coordinated single-atom catalysts (SACs) is briefly outlined in this review.
- A comprehensive summary of the recent advances on synthetic strategies and electrocatalytic applications of axially coordinated SACs is provided.
- The challenges and outlooks for future axially coordinated SACs study have been emphasized.

## Graphical abstract

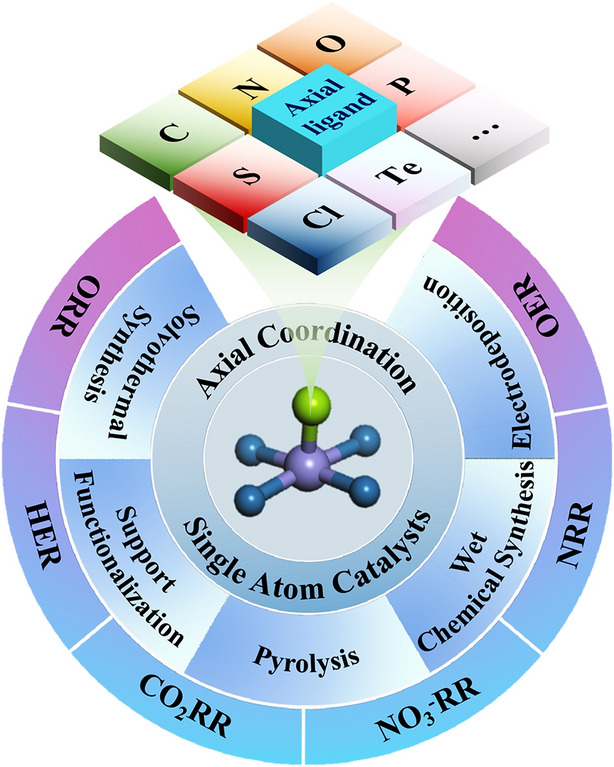

## Introduction

In the context of significant global interest in the low-carbon economy, energy issues have received intensive attention over the past decade. Despite fossil fuels currently serving as the primary energy source worldwide, their excessive utilization and various drawbacks, including land degradation, ecological deterioration, air pollution and greenhouse effect have compelled humans to explore clean and renewable low-carbon/zero-carbon energy alternatives [1, 2]. Developing sustainable and eco-friendly energy conversion technologies is crucial in addressing the growing global demand for energy while mitigating environmental concerns, including fuel cells, metal-air batteries, water electrolysis, CO_2_ reduction to value-added chemicals and/or fuels, and N_2_ fixation for ammonia synthesis [3, 4]. Electrochemical reactions such as oxygen reduction reaction (ORR) [5, 6], hydrogen evolution reaction (HER) [7, 8], oxygen evolution reaction (OER), CO_2_ reduction reaction (CO_2_RR) [9, 10], and N_2_ reduction reaction (NRR) [11–13] catalyzed by electrocatalysts have seen a series of advances in these technologies [14]. Electrocatalysts play a pivotal role in these electrochemical processes [15], as they can significantly accelerate the reaction kinetics by reducing the energy barriers and altering reaction pathways, thereby improving overall energy conversion efficiency.

Developing efficient electrocatalysts is a crucial determinant for the future industrial-scale implementation of electrochemical reactions in energy conversion and storage technologies [16]. In recent years, the high efficiency of numerous electrocatalysts has been confirmed, including platinum (Pt) group metals (PGMs) [17], carbon-based metal-free catalysts [18], single-atom catalysts (SACs) [19] and so on. PGM-based catalysts exhibit superior catalytic performance, yet their exorbitant cost and limited availability impede their further development and application [20]. Despite the abundance and structural versatility of carbon-based metal-free catalysts, there remains lack of understanding regarding their doping effects and structural diversity [21]. Since Qiao et al. [22] discovered Pt_1_/FeO_x_ SAC for CO oxidation in 2011, transition metal-based SACs have been extensively investigated for their remarkable electrocatalytic activity and promising development prospects. Particularly those transition metal-based SACs loaded on nitrogen (N)-doped carbon (M–N–C) [23], benefiting from their unique electronic and geometric structures as well as maximum atom utilization efficiency, they could exhibit diverse adsorption behaviors, and thus adjustable catalytic performance [24]. For instance, Wan et al. [25] demonstrated the application of concave Fe–N–C SAC for ORR catalysis in fuel cells, which has successfully met the activity target set by the United States in 2018 without resorting to a PGM catalyst. Currently, research on M–N–C type SACs has become a hot topic in electrocatalysis [26–28].

As widely recognized, the catalytic performance of SACs is determined by the intrinsic activity of their central atoms [29]. Specifically, the adsorption of active intermediates is constrained by the inherent properties of a single atomic site [30] and surface electronic structure [31]. Therefore, various types of metals have been tested as the atomic center, including conventional PGMs such as Pt and non-PGMs represented by Fe, Co and Ni [32]. In addition, the electrocatalytic activity of SACs can be further enhanced by precisely fine-tuning their structure [33], such as exploiting the interaction between metal atoms and supports, heteroatoms, and even adjacent metal atoms [34]. Up to now, numerous studies have been conducted to optimize the local structure of SACs for enhanced electrocatalytic performance [35–37]. Various methods including coordination engineering, heteroatom doping, construction of double or multiple atomic sites, support structure regulation, and catalytic site coupling have been proposed to regulate SACs [38, 39]. Among them, coordination engineering has been extensively studied in recent years as an effective approach to enhance the performance of SACs [40]. For instance, Yang et al. [41] prepared Mn-SAC with dual O and N atom coordination (Mn–N_3_O_1_) which demonstrated superb ORR performance. In the active site of Mn–N_3_O_1_, the coordination of O and N atoms tunes the *d-band* electronic structure of Mn to the optimal state, thereby exhibiting the fastest ORR kinetics. Guided by density functional theory (DFT) calculations, Chen et al. [42] synthesized Co_1_–N_3_PS/HC SAC with single Co atom coordinated to N, P and S, which exhibited excellent ORR activity with a half-wave potential (*E*_1/2_) of 0.920 V in alkaline medium. Besides, in electrocatalytic CO_2_ reduction, the optimized Co–N_2_C_2_ active sites displayed higher activity and selectivity compared to the prototypical Co–N_4_ active sites [43]. In OER electrocatalysis, a single Ni atom coordinated with either O or both N and S exhibited superior performance compared to traditional Ni–N_4_ sites [44, 45]. At the same time, with the development of characterization techniques such as spherical aberration-corrected high-angle annular dark field scanning transmission electron microscopy (AC HAADF-STEM), X-ray photoelectron spectroscopy (XPS), synchrotron radiation-based X-ray absorption spectroscopy (X-ray absorption structure spectroscopy (XANES) and extended X-ray absorption spectrum fine structure (EXAFS) and in combination with DFT simulation, the exact position and geometric structure of single atoms, the valence state and the atomic coordination environment can be clearly identified [46], thus promoting the knowledge about coordination regulation principle and gaining understanding of the structure–activity interplay in SACs.

**Fig. 1:**
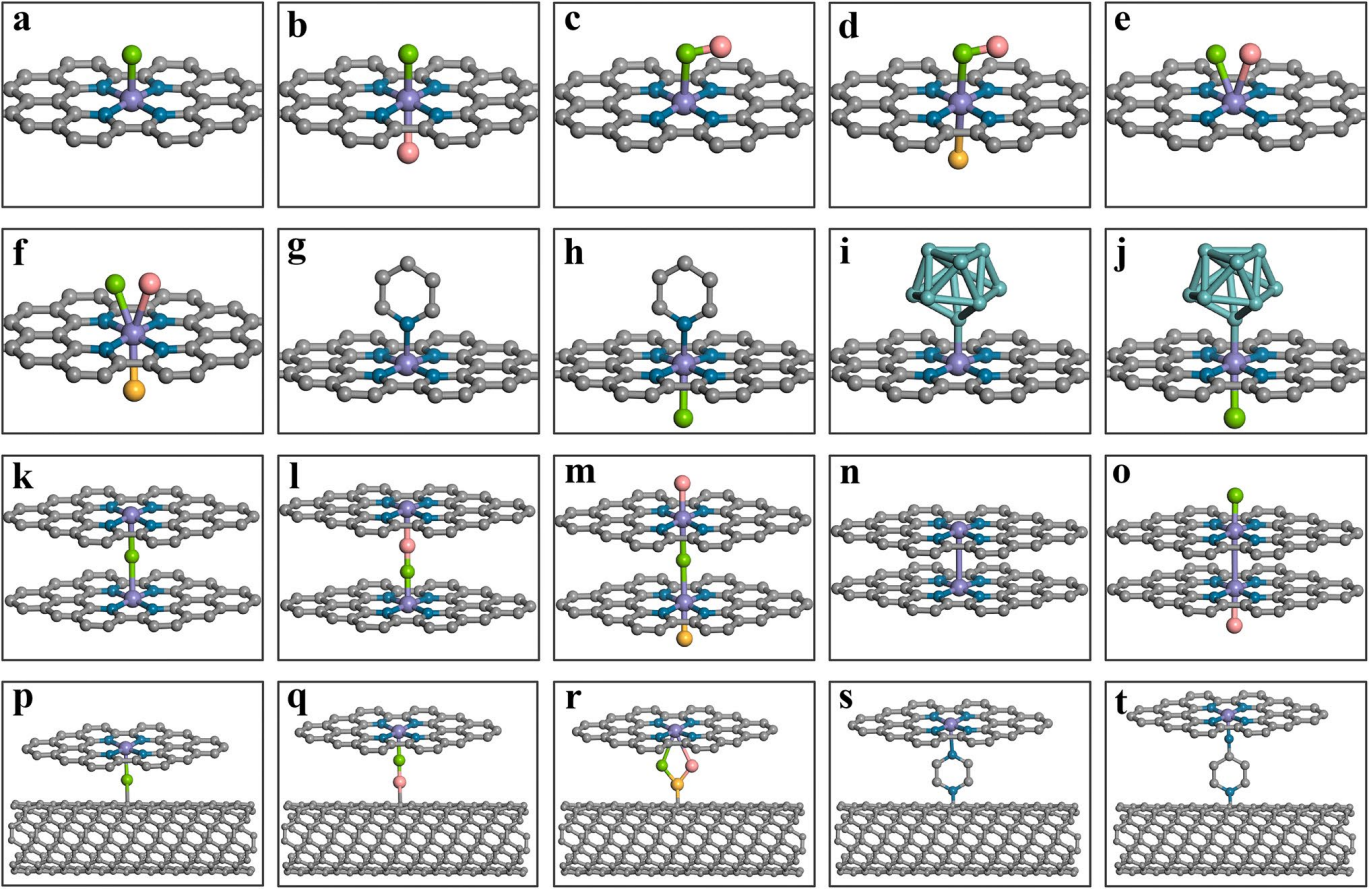
Representative and proposed possible configurations of axial ligand (denoted by letter X) coordinated MN_4_ SACs. **a** MN_4_ SAC with one axial ligand (X–MN_4_). **b** MN_4_ SAC with two axial ligands in opposite direction (X_1_–MN_4_–X_2_). **c** MN_4_ SAC with one axial group ligand (X_1_–X_2_–MN_4_). **d** MN_4_ SAC with one axial ligand and one axial group ligand in opposite direction (X_1_–X_2_–MN_4_–X_3_). **e** MN_4_ SAC with two axial ligands in the same direction (X_1_/X_2_–MN_4_). **f** MN_4_ SAC with two axial ligands in the same direction and one axial ligand in the opposite direction (X_1_/X_2_–MN_4_–X_3_). **g** MN_4_ SAC with one axial pyridine ligand (Py–MN_4_). **h** MN_4_ SAC with one axial pyridine ligand and one axial ligand in opposite direction (Py–MN_4_–X). **i** MN_4_ SAC with one axial metal cluster ligand (M_cluster_–MN_4_). **j** MN_4_ SAC with one axial metal cluster ligand and one axial ligand in the opposite direction (M_cluster_–MN_4_–X). **k** Two MN_4_ SACs bridged by one axial ligand in between (MN_4_–X–MN_4_). **l** Two MN_4_ SACs bridged by two axial ligands in between (MN_4_–X_1_–X_2_–MN_4_). **m** Two MN_4_ SACs bridged by one axial ligand in between and with one axial ligand in each of the opposite direction (X_2_–MN_4_–X_1_–MN_4_–X_3_). **n** Two MN_4_ SACs axially bridged by their central metal atoms (MN_4_–MN_4_). **o** Two MN_4_ SACs axially bridged by their central metal atoms and with one axial ligand in each of the opposite direction (X_1_–MN_4_–MN_4_–X_2_). **p** MN_4_ SAC with one axial ligand grafted on CNT support (MN_4_–X–CNT). **q** MN_4_ SAC with one axial group ligand grafted on CNT support (MN_4_–X_1_–X_2_–CNT). **r** MN_4_ SAC with two axial ligands in the same direction and co-grafted on CNT support (MN_4_–X_1_/X_2_–X_3_–CNT). **s** MN_4_ SAC with one axial pyrazine ligand grafted on CNT support (MN_4_–Pyr–CNT). **t** MN_4_ SAC with one axial 4-pyridylamine ligand grafted on CNT support (MN_4_–NH_2_–Py–CNT)

Over the past two years, inspired by the active iron sites of natural enzymes like horseradish peroxidase (HRP) and cytochrome c oxidases (CcO), Fe–N–C-based SACs with penta-coordinated heme-like active site structure (Fe–N_5_–C) have attracted great attention in electrocatalysts [47–51]. The fifth N ligand in the axial direction could exert an electron pull–push effect and/or steric effect on the single-atom sites, which modulates the binding strength between the active sites and reaction intermediates and consequently enhanced the activity of the axially modified SACs. In this background, axial coordination engineering is proposed to be a new coordination tuning method for tuning local coordination structure of the single-atom sites in SACs. In contrast to typical M–N_4_ SACs, the axial coordination design will bring forth new types of coordination configurations that hold great potential for achieving significantly boosted catalytic activity in certain active site-specific catalysis applications. By introducing one or multiple additional ligands in the axial position perpendicular or non-coplanar to the planar M–N_4_ sites in SACs, it is anticipated that the electron distribution symmetry will be disrupted and the electronic structure of the central single-atom active sites will be efficiently altered, thus optimizing the adsorption behavior and decreasing energy barriers for the intermediate sorption. Moreover, the axial ligand introduced over the central single-atom sites could alternatively serve as the new adsorption site to synergize with the M–N_4_ site for catalysis, ultimately changing the reaction pathways to be more energetically favorable. To date, various ligands such as N-containing ligands [52] (N, NH_2_, macrocyclic compounds, etc.), O-containing ligands [53] (O, OH, etc.), halogen-containing ligands [54] (Cl, Br, I), carbon-containing ligands (C, CNT, graphene, etc.), metal-containing ligands (PtO_2_, Te cluster, etc.) and other types of ligands have been introduced to the axial position of SACs. Figure 1 depicts the recently reported as well as the potential axial coordination configurations of SACs based on the classical planar MN_4_ configuration. The axial coordination could significantly alter the electrocatalytic performance of SACs by introducing external atoms, functional groups, and even molecules into the metal atom center of SACs [55]. Through the axial coordination design of SACs, novel properties are conferred upon the central metal atom, thereby enabling regulation of the electrocatalytic activity, selectivity and stability of SACs. Generally, axial ligands serve three primary functions: (1) anchoring functional complex onto the electrode surface; (2) serving as molecular wires to facilitate electron transport between the electrode and metal center; (3) modifying the electron density of the metal center and altering its reactivity [56]. In this review, we will focus on the discussion of the recent development of axial coordination modification of SACs for electrocatalytic applications in energy conversion. We first provide a comprehensive summary of various synthetic strategies employed for achieving axial-coordinated SACs. Furthermore, we present a comprehensive demonstration of the efficacy of axial coordination design of SACs in energy electrocatalysis, encompassing a range of reactions including ORR, CO_2_RR, HER, OER, NRR and beyond.

## Synthetic Strategies of Axial Coordination Design of SACs

Due to the high surface energy of atoms, aggregation and deactivation are prone to occur during the preparation and catalytic processes, which imposes higher requirements on the synthetic process of SACs [57]. Therefore, reviewing the synthetic strategies is instrumental in establishing a theoretical basis for innovative endeavors aimed at proposing more efficient approaches in the future. So far, many strategies have been developed for the synthesis of axially coordinated SACs, with high-temperature pyrolysis being the most commonly employed method. However, precise control of catalyst structure is challenging due to the intricate reaction mechanisms at elevated temperatures. Therefore, alternative methods have been further developed and provide a solid foundation for constructing axially coordinated SACs with well-defined structures. In this section, we present a summary of the latest synthetic strategies for designing axially coordinated SACs, including high-temperature pyrolysis, solvothermal synthesis, wet chemical synthesis, support functionalization method, and electrodeposition method.

### High-Temperature Pyrolysis

**Fig. 2:**
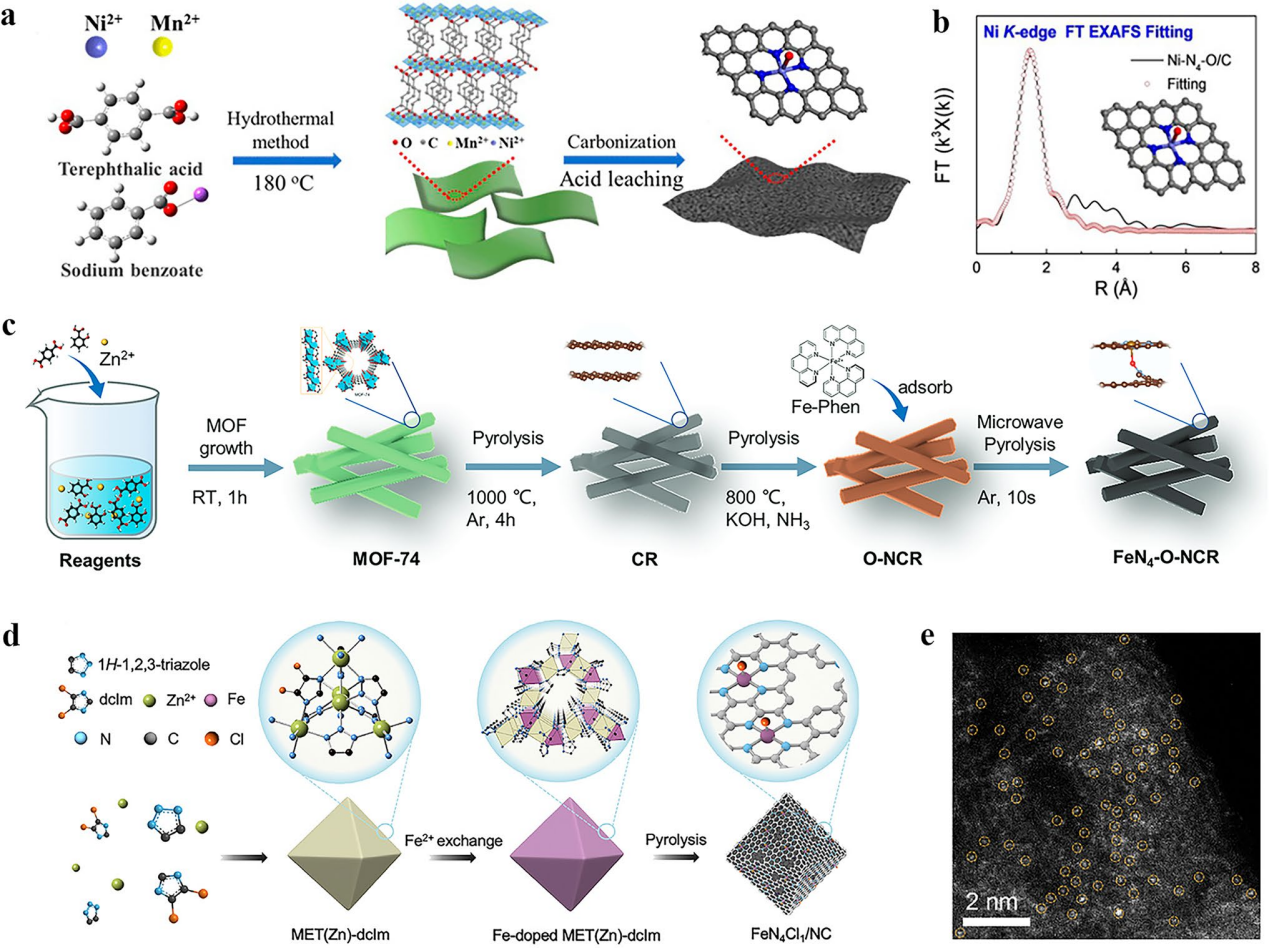
Synthesis of SACs with axial coordination by high-temperature pyrolysis. **a** Illustration of the synthetic process of Ni–N_4_–O/C. **b** EXAFS fitting curve of Ni–N_4_–O/C in R space, inset is the corresponding schematic model of Ni–N_4_–O/C. Reproduced with permission [60]. Copyright: 2020, Wiley-VCH GmbH. **c** Schematic illustration of the synthesis of FeN4–O–NCR catalyst. Reproduced with permission [61]. Copyright: 2022, Wiley-VCH GmbH. **d** Preparation route and **e** AC HAADF-STEM image of FeN_4_Cl_1_/NC. Reproduced with permission [64]. Copyright: 2022, Wiley-VCH GmbH

High-temperature pyrolysis is a commonly employed method for the preparation of axially coordinated SACs, involving calcination of precursors under an inert atmosphere at temperatures ranging from 600 to 1000 °C [58]. Xiao et al. [59] successfully prepared atomically dispersed Fe–N–C SAC through a one-step pyrolysis of the ZIF-8 precursor. During this process, Fe atoms were immobilized within the N-doped porous carbon skeleton through coordination with four N atoms in the plane and two O atoms in the axial direction. Similarly, Ni–N_4_–O/C catalysts with O axial coordination were synthesized by Wang et al. [60] via direct carbonizing of the precursor followed by acid etching (Fig. 2a). Through EXAFS curve fitting analysis of Ni–N_4_–O/C, precise structural parameters surrounding the central Ni atom were obtained, which further elucidated that the coordination configuration of single Ni atom adopts a planar Ni–N_4_ configuration with one axial Ni–O bond (Fig. 2b). Besides direct high-temperature pyrolysis, stepwise pyrolysis is also a viable and important method for preparing SACs. Peng et al. [61] initially subjected the MOF-74 precursor to pyrolysis at 1000 °C in an Ar atmosphere, resulting in the formation of one-dimensional carbon nanorods. Subsequently, they employed a molten salt (KOH) assisted pyrolysis strategy under NH_3_ atmosphere to prepare O, N-doped carbon nanorods (O–NCR). The Fe-phenanthroline complex was then electrostatically adsorbed onto the O-NCR, and rapid microwave-assisted carbonization was performed to yield the final axially coordinated FeN_4_–O–NCR catalyst (Fig. 2c). The Fe K-edge X-ray absorption spectroscopy (XAS) was employed to determine the detailed atomic structures of FeN_4_–O–NCR and FeN_4_/CR. The Fe K-edge XANES spectra of both samples exhibit a close resemblance to that of iron phthalocyanine (FePc), indicating the presence of cationic Fe states in the sample. The fitting results indicate that the average oxidation state of Fe in FeN_4_–O–NCR (+2.7) is higher than that in FeN_4_/C (+2.4). This higher average valence of Fe in FeN_4_–O–NCR is consistent with the presence of an additional axial O ligand bound to Fe. The average Fe–N distances of FeN_4_–O–NCR and FeN_4_/CR in R space are 1.97 and 1.99 Å, respectively, indicating that the planarity of FeN_4_ moiety is not significantly altered by the presence of axial O ligand.

However, the preparation of axially coordinated SACs may not always be achieved by a simple high-temperature pyrolysis method alone, which has prompted researchers to explore its combination with other techniques. For example, after synthesizing Fe/N-G-800 catalysts through high-temperature pyrolysis at 800 °C, Xu et al. [62] employed impregnation method to anchor FePc molecules onto the Fe/N-G-800 matrix via axial coordination, resulting in the final product of Fe@Fe/N-G-800 catalyst. The XAS measurements were utilized to investigate the chemical environment of Fe atoms in Fe@Fe/N-G-800, revealing that the oxidation state of Fe is between + 2 and + 3 based on the Fe K-edge XANES results. Besides, EXAFS spectroscopy results confirmed that the Fe species in Fe/N-G-800 predominantly exist in the atomically dispersed Fe–N_4_ and Fe–O configurations, with coordination numbers of 3.8 and 1.0, respectively. Therefore, an axial O bridge bond of Fe–O–Fe and an O–FeN_4_ structure were proposed. In the course of synthesizing Sn–N–C SAC with O axial coordination, Luo et al. [63] employed multiple pyrolysis steps in conjunction with additional processes of reflux and ball milling. Prior to high-temperature pyrolysis of the precursor for obtaining the axial Cl-coordinated FeN_4_Cl_1_/NC catalyst, Hu et al. [64] employed a post-synthetic ion-exchange technique to substitute partial Zn^2+^ ions in MET (Zn)-dcIm nanocrystals with Fe^2+^ ions, which facilitated the subsequent pyrolysis (Fig. 2d, e).

### Solvothermal Synthesis

**Fig. 3:**
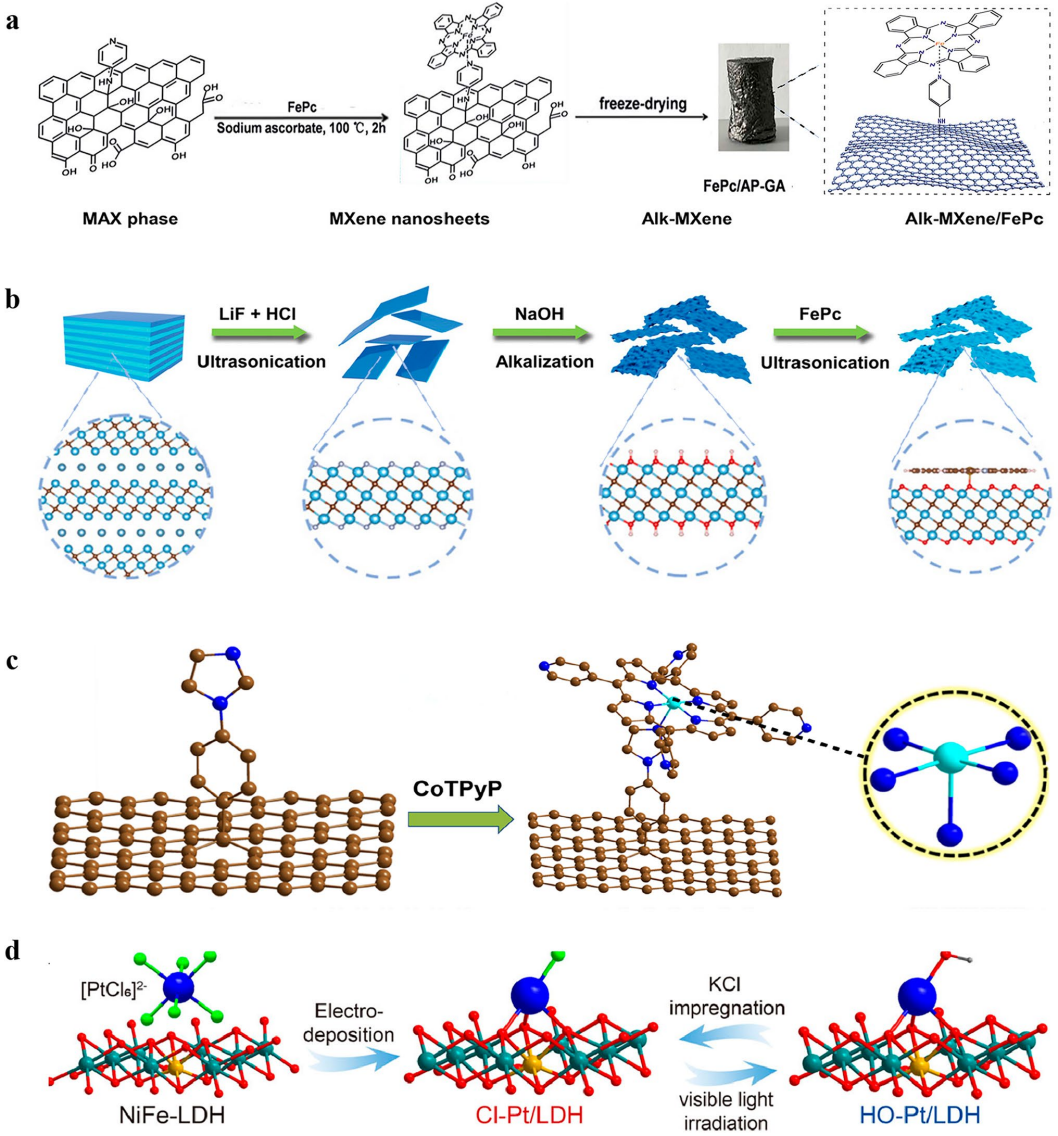
**a** Fabrication process of the 3D FePc/AP-GA catalyst by solvothermal synthesis. Reproduced with permission [65]. Copyright: 2018. The Royal Society of Chemistry. **b** Schematic synthesis of Alk-MXene/FePc catalyst by wet chemical synthesis. Reproduced with permission [68]. Copyright: 2023, Wiley-VCH GmbH. **c** Schematic illustration of the synthesis of CoTPyP@Im-RGO by support functionalization method. Reproduced with permission [71]. Copyright: 2022, Elsevier Ltd. **d** Schematic illustration for the synthesis of Cl-Pt/LDH catalyst by electrodeposition method and the following reversible axial ligand exchanging procedures. Atoms are indicated by spheres: Pt (blue), Ni (olive), Fe (yellow), O (red), Cl (green), and H (gray). Reproduced with permission [77]. Copyright: 2022, Springer Nature. (Color figure online)

Due to its ease of operation, simplicity of procedures, and high level of safety compared with the high-temperature pyrolysis method, solvothermal method has gained widespread popularity for synthesizing SACs. Therefore, researchers are dedicated to employing this approach for the production of axially coordinated SACs. Of course, in order to synthesize axially coordinated SACs and not just SACs, hydrothermal precursors must use materials that can perform the fifth coordination, such as FePc. Huang et al. [65] synthesized FePc/AP GA composites using a one-step hydrothermal method. In this process, both the loading of FePc and the formation of 3D graphene hydrogel were achieved (Fig. 3a). An aqueous solution of FePc in tetrahydrofuran and sodium ascorbate were added to the AP-GO dispersion, followed by reaction at 100 °C for 2 h in a high-pressure reactor. The molded graphene hydrogel was then extracted using tweezers and soaked in deionized water for two days to eliminate any residue or weakly adsorbed FePc. Finally, the hydrogel underwent freeze-drying to yield FePc/AP-GA. The functional groups present in the products were initially analyzed using Fourier transform infrared spectroscopy (FTIR). The results indicate that FePc molecules were anchored to the graphene surface via coordination interaction with the axial 4-aminopyridine (4-AP) linker. Although solvothermal synthesis of SACs is relatively common, the synthesis of SACs with axial coordination structure by this method is not yet widespread. Therefore, the development of efficient solvothermal method to precisely engineer axial ligands in SACs is highly desired.

### Wet Chemical Synthesis

The wet chemical synthesis is a commonly employed method for synthesizing SACs, including those with axial coordination structures. This synthetic approach involves the thorough mixing of metal salts with appropriate supports, gradual adsorption of metal ions onto the surface or pore structure of the supports, and subsequent large-scale production of SACs through drying and reduction processes [66]. Different from the simple wet chemical synthesis of conventional SACs, in the synthetic processes of axially coordinated SACs, the metal precursors impregnated on the support basically contains M–N_4_ configuration, or the supports themselves have the M–N_4_ moiety, which is also a prerequisite for the formation of axial coordination configuration of SACs. For example, Zhao et al. [67] employed a facile wet-impregnation method to synthesize Pt-ACs/CoNC featuring axial Co–O–Pt bonding, in which the Pt atomic clusters were axially anchored onto Co–N–C single atom sites. Besides, Dai et al. [68] treated the Ti_3_C_2_T_x_ MXene nanosheets in NaOH solution to generate alkaline Ti_3_C_2_(OH)_x_ MXene (Alk MXene), which was then combined with FePc via ultrasonic mixing, producing an Alk-MXene/FePc hybrid catalyst featuring an Fe–N_4_O_1_–OC quasi configuration (Fig. 3b). To further elucidate the local coordination geometry and electronic states, XANES and EXAFS analyses were conducted. The Fe K-edge of Alk-MXene/FePc falls between FeO and Fe_2_O_3_, indicating the oxidation state of Fe is between + 2 and + 3. The FT-EXAFS spectra of FePc and Alk-MXene/FePc exhibit main peaks at 1.41 and 1.50 Å in R-space, respectively. The increased height of the main peak confirms the presence of an axial Fe–O bond within the first coordination sphere. Furthermore, the WT-EXAFS contour maps reveal a maximum intensity of 7.6 Å^–1^ for FePc and 6.9 Å^–1^ for Alk-MXene/FePc, suggesting that axial coordination with Fe–O may alter the coordination environment of the Fe center. Moreover, Guo et al. [69] employed the same method to synthesize Fe-SACs with axial O coordination. The FePc perchlorate (FePc‧ClO_4_) was rapidly added to the GO aqueous dispersion system in acetonitrile, followed by a reduction using NaBH_4_ and hydrazine hydrate to obtain the FePc/RGO catalysts. X-ray photoelectron spectroscopy (XPS) revealed a decrease in the binding energy of N atoms within Fe–N (N_2_, pyrrol-N) bonds and a consequent change in the coordination number of Fe atoms from 4 to 5 when compared with FePc. The Fe K-edge XANES analysis provided further insights into the structural characteristics of FePc/RGO SAC, indicating that FePc is axially coordinated to RGO via an O bridge.

### Support Functionalization Method

Support functionalization techniques are predominantly employed to tether metal phthalocyanine or porphyrin onto CNTs or graphene via intermediates such as pyridine, which simplifies the synthesis of the classical axial coordination structure of such SACs [70]. For example, Yang et al. [71] used this method to anchor a Co porphyrin molecule, CoTPyP, onto functionalized reduced GO (Im@RGO) and provided an axial ligand to the cobalt center (Fig. 3c). The synthesis involved diazotization of 4-imidazole-1-yl-aniline to reduce GO and form Im@RGO, followed by co-refluxing of CoTPyP and Im@RGO in N, N-dimethylformamide (DMF) under an N_2_ atmosphere to yield CoTPyP@Im-RGO. The electronic structure and coordination environment of CoTPyP@Im-RGO were investigated using XANES and EXAFS techniques, revealing that Co is present as a single atom with a positive charge, and the average valence state of Co ranges from + 2 to + 3. The Co active center forms both a planar Co–N_4_ structure by bonding with N atoms from porphyrin and axial coordination with N on carbon support to create a Co–N_5_ structure. This method has also been employed in other studies for the synthesis of metal phthalocyanines with axial coordination structure. Riquelme et al. [72] synthesized CoPc-Py-CNT catalysts using this method. They functionalized CNTs with pyridine (Py) through diazotization reaction to yield Py-CNT, followed by the introduction of CoPc onto the surface of Py-CNT by refluxing in N_2_ at 150 °C in DMF to yield CoPc-Py-CNT. The synthesized catalyst possesses a Co–N_5_ structure, with four N atoms forming a planar arrangement with Co and one N atom coordinating axially with Co. Later, Fan et al. [73] also synthesized CoPc-Py-CNT catalysts with axial N coordination using the same method. After grafting pyridine onto CNTs (CNT-Py), a mixture of CNT-Py and CoPc was refluxed in tetrahydrofuran (THF) for 1 h under Ar protection, thus giving rise to N-axially coordinated Co-SACs.

### Electrodeposition Method

Electrodeposition for axial coordination design of SACs is a recently developed method that involves the dissolution of bulk metal into a solution, resulting in the formation of atomic metal species that are subsequently anchored onto a support using electrochemical means [74]. During the synthesis of SACs, metal ions are transformed into metal atoms by electrodeposition and deposited as SACs onto the supports on the working electrode [75]. In contrast, in the synthesis of axially coordinated SACs, metal coordination ions transform to metal atoms while serving as an axial ligand to the SACs that already formed on the support [76]. Moreover, the metal ligand can also be replaced into a series of other ligands by certain methods. Zhang et al. [77] employed NiFe-LDH nanosheet arrays as the support, and used the electrodeposition method to synthesize the Cl–Pt/LDH catalyst (Fig. 3d). The Cl–Pt/LDH was prepared in 1 M KOH solution containing PtCl_6_^2–^ anions using a three-electrode system, with NiFe-LDH serving as the working electrode. During the process of electrolysis, PtCl_6_^2−^ anions were electrodeposited and adsorbed onto the surface of LDH to generate atomically dispersed Pt sites with axial Cl coordination. Notably, the axial ligand can be altered by a further photochemical method. Upon visible light irradiation in water, the axial Cl ligand was replaced by a hydroxide group. Noteworthily, the exchanged hydroxyl group can be reversibly switched back to the axial Cl ligand after a KCl impregnation treatment. Currently, the development of electrochemical methods for constructing axially coordinated SACs is still very limited. However, electrodeposition offers a promising avenue for the synthesis of SACs with axial coordination.

## Axial Coordination Design of SACs in Energy Electrocatalysis Applications

Due to their cost effectiveness, maximized atom utilization efficiency and highly tunable properties, the use of transition metal SACs in electrocatalysis has become a prominent research [78]. The intrinsic activity and density of metal active sites are key factors that influence the catalytic ability of SACs [79]. Consequently, various SACs featuring diverse active sites and matrices have been designed and studied. It is widely accepted that M–N–C (M = Fe, Co, Ni, Mn, Cu, etc.) structures primarily serve as active sites for SACs [80], and precisely manipulating the environmental structure surrounding metal atoms can significantly improve the electrochemical performance of catalysts [81]. In a bid to further enhance the activity of SACs, several strategies have been investigated and implemented [82, 83]. One such strategy involves changing the coordination number of central transition metal atoms from four to five or six by adding axial coordination ligands, which alters the coordination environment and electronic structure of the central atom and allows for the regulation of electrocatalytic performance of SACs. To date, reported axial coordination designs of SACs used in electrocatalytic reactions can be categorized into five categories based on the axial ligands used: (1) nitrogen-containing ligands, (2) oxygen-containing ligands, (3) sulfur-containing ligands, (4) halogen-containing ligands, and (5) other ligands. In the following section, we focus on discussion of the electrocatalytic performance of SACs coordinated with the above axial ligands in various energy-related electrochemical reactions.

### Oxygen Reduction Reaction

The oxygen reduction reaction (ORR) is an important and extensively studied electrochemical reaction [84]. It is a multistep process and generally involves two distinct reaction pathways, depending on the number of electron transferred [85]. Specifically, O_2_ molecules can be reduced to either H_2_O through a four-electron (4e^–^) reaction pathway (Eqs. (1) and (2)) or to H_2_O_2_ through a two-electron (2e^–^) reaction pathway (Eqs. (3) and (4)) [86].

4e^–^ processes:

In acidic medium (pH = 0):

1 $${\text{O}}_{{2}} + {\text{4H}}^{ + } + {\text{4e}}^{ - } \to {\text{2H}}_{{2}} {\text{O}},    E^{^\circ } = {1}.{23}\;{\text{V}}\;\;{\text{vs}}.{\text{ SHE}}$$

In alkaline medium (pH = 14):

2 $${\text{O}}_{{2}} + {\text{2H}}_{{2}} {\text{O}} + {\text{4e}}^{ - } \to {\text{4OH}}^{-} , E^{^\circ } = 0.{4}0 \;{\text{V}}\;\;{\text{vs}}.{\text{ SHE}}$$

2e^–^ processes:

In acidic medium (pH = 0):

3 $${\text{O}}_{{2}} + {\text{2H}}^{ + } + {\text{2e}}^{ - } \to {\text{H}}_{{2}} {\text{O}}_{{2}}, E^{^\circ } = 0.0{6}\; {\text{V vs}}.{\text{ SHE}}$$

In alkaline medium (pH = 14):

4 $${\text{O}}_{{2}} + {\text{2H}}_{{2}} {\text{O}} + {\text{2e}}^{ - } \to {\text{H}}_{{2}} {\text{O}}_{{2}} + {\text{2OH}}^{ - }, E^{^\circ } = 0.{7}0\; {\text{V}}\;\;{\text{vs}}.{\text{ SHE}}$$

The 4e^–^ reaction pathway in electrochemical ORR is composed of two distinct mechanisms (Eqs. (5) and (6)). The 2e^–^ reaction pathway includes only one *OOH reaction intermediate (Eqs. (7)). The ORR reaction mechanism is illustrated below [87]:

4e^–^ processes:

5 $${\text{O}}_{{2}} + {4}\left( {{\text{H}}^{ + } + {\text{e}}^{ - } } \right) \to *{\text{OOH}} + {3}\left( {{\text{H}}^{ + } + {\text{e}}^{ - } } \right) \to *{\text{O}} + {2}\left( {{\text{H}}^{ + } + {\text{e}}^{ - } } \right) \to *{\text{OH}} + {1}\left( {{\text{H}}^{ + } + {\text{e}}^{ - } } \right) \to {\text{H}}_{{2}} {\text{O}}$$

6 $${\text{O}}_{{2}} + {4}\left( {{\text{H}}^{ + } + {\text{e}}^{ - } } \right) \to {2}*{\text{OH}} + {2}\left( {{\text{H}}^{ + } + {\text{e}}^{ - } } \right) \to {\text{H}}_{{2}} {\text{O}}$$

2e^–^ processes:

7 $${\text{O}}_{{2}} + {2}\left( {{\text{H}}^{ + } + {\text{e}}^{ - } } \right) \to *{\text{OOH}} + {1}\left( {{\text{H}}^{ + } + {\text{e}}^{ - } } \right) \to {\text{H}}_{{2}} {\text{O}}_{{2}}$$

Both 4e^–^ ORR and 2e^–^ ORR processes have significant importance in scenes of daily life and industrial chemicals synthesis. The 4e^–^ transfer process in the cathodic ORR reaction is specifically desirable for proton-exchange membrane fuel cells (PEMFCs) and metal-air batteries [85]. However, due to the sluggish reaction kinetics of ORR, even the most efficient Pt-based catalysts require a high Pt loading to attain an optimal fuel cell performance [88]. As a consequence, extensive research has been carried out to develop cost-effective and available electrocatalysts for PEMFCs, encompassing advanced Pt alloys, heteroatom-doped nanocarbons, SACs and so on [89]. SACs are a kind of important electrocatalysts to remarkably improve the performance of ORR, and various strategies have been developed to regulate the active site structures of SACs [90, 91]. For instance, Yuan et al. [92] reported a Co-SAC featuring abundant carbon defects, which can significantly decrease the adsorption free energy of *OOH on Co–N_4_ sites, thereby enhancing the ORR catalytic performance. Zhang et al. [93] prepared an S-doped FeN_3_S active site and evaluated its electrochemical ORR performance. Their findings revealed that the FeN_3_S SAC exhibited exceptional ORR performance after S doping, potentially due to the optimized charge and spin distribution in Fe–N–C materials.

Additionally, another significant application of ORR is the generation of H_2_O_2_ through the 2e^–^ pathway. As one of the most crucial chemicals, H_2_O_2_ has a vast array of industrial applications, such as chemical synthesis [94], pulp and paper bleaching [95], wastewater treatment [96], and others. The current industrial method for producing H_2_O_2_ is primarily the anthraquinone oxidation process, which suffers from drawbacks such as high energy consumption, complex infrastructure, and significant waste generation [97]. In recent years, the electrochemical synthesis of H_2_O_2_ from ORR through 2e^–^ transfer process has been extensively investigated due to its advantages of mild reaction conditions and pollution-free waste [98]. The utilization of SACs also confers significant benefits in facilitating 2e^–^ ORR. In principle, to achieve highly selective synthesis of H_2_O_2_ via 2e^–^ ORR, it is essential to avoid O–O bond cleavage. Due to the unique structure of SACs, where metal centers are atomically dispersed, O_2_ adsorption on SACs typically follows Pauling-type coordination rather than side-on coordination, which reduces the feasibility of O–O bond breaking [99]. Therefore, SACs are promising candidates for H_2_O_2_ synthesis via 2e^–^ ORR.

Axial coordination has emerged as an effective means of regulating the structure of SACs, holding great promise in modulating their performance in ORR. To date, SACs modified with various axial ligands including N-containing, O-containing, S-containing, halogen-containing ligands and other ligands have been investigated for their ability to regulate the ORR properties. Table 1 summarizes the recent axial coordination designs of SACs for ORR.

**Table 1:** Summarized electrocatalytic ORR activity of typical axial coordination modified SACs

Axial-coordinated SACs	Coordination structure	Axial atom	Electrolyte	Pathway	*E*_1/2_ (V vs RHE)	H_2_O_2_ yield (%)	Refs
FePc/CNT-R	FeN_4_–NH_2_	N	0.1 M KOH	4e^−^	0.92	< 3	[109]
FePc–Py–CNTs	FeN_4_–Py	N	0.1 M KOH	4e^−^	0.915	< 1.5	[110]
FePc/NGM-0.25	FeN4–N	N	0.1 M KOH	4e^−^	0.90	< 1.25	[111]
Fe-SAC/N–C	FeN_4_–N	N	0.1 M KOH	4e^−^	0.89	< 1	[115]
Fe–N–C/rGO	FeN_4_–N	N	0.1 M KOH	4e^−^	0.90	< 5	[116]
Mn–NC-SA-950	MnN_4_–N	N	0.1 M KOH	4e^−^	0.852	< 5	[117]
Fe@Fe/N-G-800	FeN_4_–O	O	0.1 M KOH	4e^−^	0.866	< 3	[62]
O–Zr–N–C	ZrN_4_–O	O	0.1 M KOH	4e^−^	0.91	< 8	[120]
Co-SA@N-CNFs	CoN_4_–O	O	0.1 M KOH	4e^−^	0.85	< 20	[121]
V–N_1_O_4_	V–O_3_N_1_–O	O	0.1 M KOH	4e^−^	0.865	< 10	[122]
o-MQFe	FeN_3_O–O–Ti	O	0.1 M KOH	4e^−^	0.861	< 5	[123]
FeAB–O	FeN_4_–O	O	0.1 M KOH	4e^−^	0.90	< 1	[124]
N_4_Ni_1_O_2_/OCNTs	NiN_4_–2O	O	1.0 M KOH	2e^−^	0.68	> 90	[126]
Fe–N/S–C	Fe–N_3_S_1_OH	O	0.1 M KOH	4e^−^	0.882	< 3.2	[127]
Fe (Zn)–N–C	HO–FeN_4_–O–FeN_4_–OH	O	0.1 M HClO_4_	4e^−^	0.83	< 7	[128]
S1–Cr1N4–C	Cr_1_N_4_–S	S	0.1 M KOH	4e^−^	0.90	< 12	[129]
S-modified Fe–N–C	FeN_4_–S	S	0.1 M KOH	4e^−^	0.88	< 7	[130]
Co–ZIFs-60	CoN_4_–S	S	0.5 M H_2_SO_4_	4e^−^	0.793	< 2.2	[131]
FeN_4_Cl_1_/NC	FeN_4_–Cl	Cl	0.1 M KOH	4e^−^	0.91	< 4	[64]
Fe–N/C-SAC	FeN_4_–Cl	Cl	0.1 M KOH	4e^−^	0.91	< 6	[132]
FeCl_1_N_4_/CNS	FeN_4_–Cl	Cl	0.1 M KOH	4e^−^	0.921	< 1	[133]
Fe^Zn^/CNP (1)	FeN_4_–Cl	Cl	0.1 M KOH	4e^−^	0.88	< 10	[134]
FeN_4_Cl SAC	FeN_4_–Cl	Cl	0.1 M HClO4	4e^−^	0.818	< 1	[135]
YN_4_–Cl catalyst	YN_4_-Cl	Cl	0.1 M KOH	4e^−^	0.85	–	[137]
FePc–RCNTs	FeN_4_–C	C	0.1 M KOH	4e^−^	0.86	< 2	[139]
FeNPC	FeN_4_–PO_4_	P	0.1 M KOH	4e^−^	0.88	< 5.6	[144]
Pt_1_@Fe–N–C	FeN_4_–PtO_2_	PtO_2_	0.5 M H_2_SO_4_	4e^−^	0.80	< 2	[145]
FeN_4_–Te_n_	FeN_4_–Te_n_	Te	0.1 M KOH	4e^−^	0.867	–	[146]

#### Nitrogen (N) Ligand Axially Coordinated SACs for ORR

Since the mid-1960s [100], metal macrocyclic compounds such as metal phthalocyanine have been extensively investigated in the field of electrocatalysis. Because their well-defined M–N_4_ configuration could serve as an excellent model for investigating the ORR mechanism and regulating the catalytic activity of SACs [101]. However, metal macrocyclic compounds lack long-term stability [102], making them inadequate for practical electrocatalytic applications. To address this challenge, researchers have explored various approaches, such as modifying the chemical structure and incorporating functional groups, atoms, or various substrates, to modulate the catalytic activity of metal macrocycles [103].

**Fig. 4:**
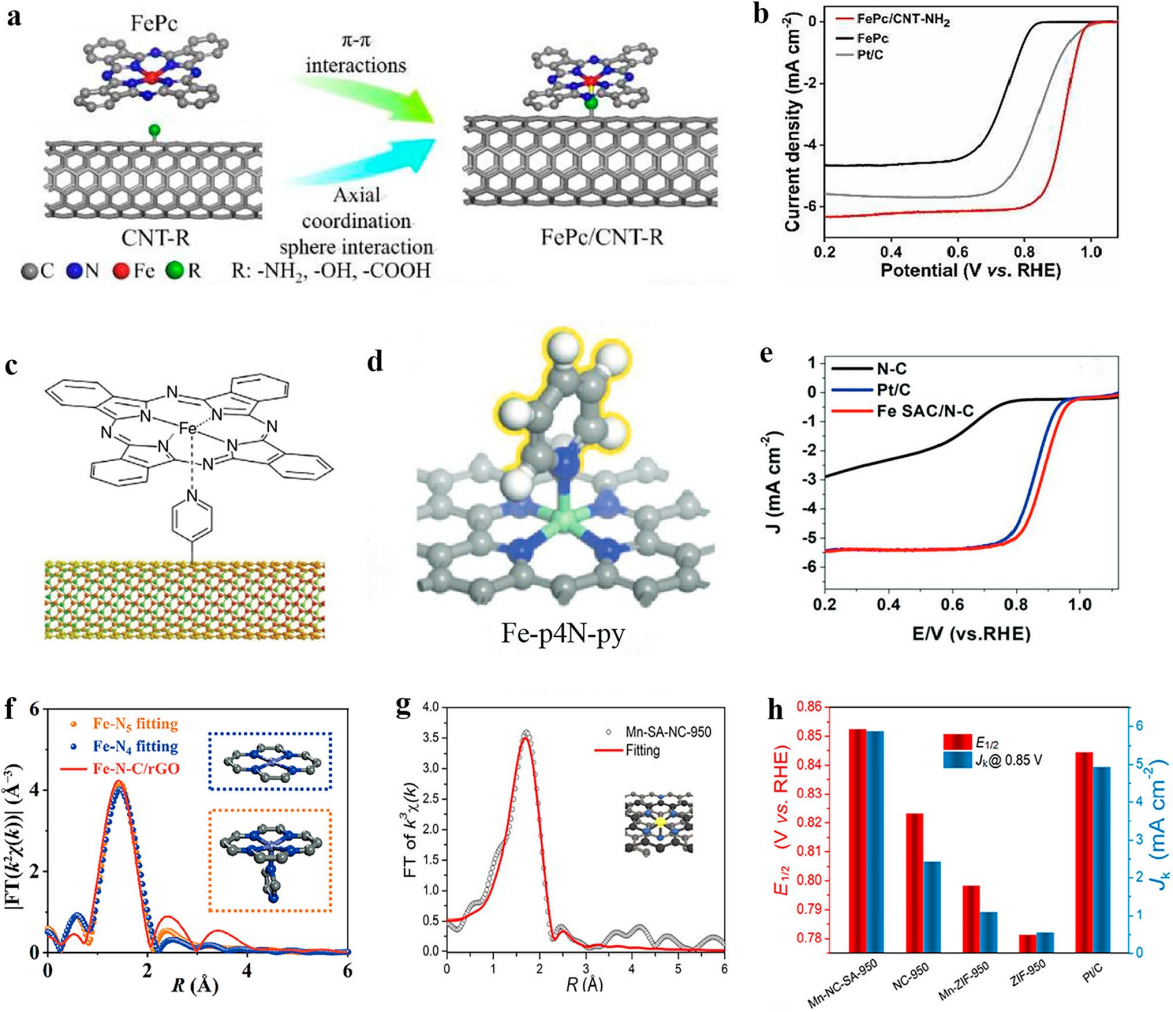
**a** Schematic representation of the self-assembly process of the FePc/CNT–R catalyst. **b** LSV curves of FePc, FePc/CNT–NH_2_, and Pt/C in O_2_-saturated 0.1 M KOH. Reproduced with permission [109]. Copyright: 2021 Wiley-VCH GmbH. c Schematic diagram of the structure of FePc–Py–CNTs composite. Reproduced with permission [110]. Copyright: 2013, Nature Publishing Group. **d** Top view of Fe-SACs models with Fe–p4N–py coordination configuration. **e** LSV curves of Fe-SAC/N–C, N–C, and commercial Pt/C in O_2_-saturated 0.1 M KOH with a sweep rate of 10 mV s^−1^ and a rotating rate of 1600 rpm. Reproduced with permission [115]. Copyright: 2019 WILEY-VCH Verlag GmbH & Co. KGaA, Weinheim. **f** R space fitting curve of Fe–N_4_ configuration (marked by blue dotted frame) and Fe–N_5_ configuration (marked by orange dotted frame, here hydrogen atoms on the imidazole ring are hidden to make the structure more visible). Reproduced with permission [116]. Copyright: 2022 Springer Nature. **g** Mn K-edge EXAFS fitting curve at R space of Mn-NC-SA-950. **h** *E*_1/2_ and *J*_k_ at 0.85 V of the prepared catalysts and Pt/C catalyst in O2-saturated 0.1 M KOH solution at a scan rate of 10 mV s^−1^. Reproduced with permission [117]. Copyright: 2023, ELSEVIER B.V. and Science Press

Graphene [104], carbon nanotubes (CNTs) [105, 106], and carbon nanofibers [107] are known to exhibit excellent catalytic activity for ORR. Recently, electrocatalysts comprising of metal phthalocyanine with pyridine or imidazole fixed on carbon nano-materials have gained increasing attention due to their low ORR overpotential [108]. The axial coordination of electron-donating ligands onto metal phthalocyanine facilitates the adsorption of O_2_ molecules and switches the formation of *OOH intermediates to be the rate-limiting step. In addition, the re-hybridization of Fe 3d-orbitals with axially coordinated ligand orbitals leads to significant alterations to both electronic and geometric structures, thereby greatly enhancing the rate of ORR. Zhang et al. [109] have demonstrated that the electrochemical ORR performance of functionalized multi-walled CNTs (CNT-R, R = NH_2_, COOH, or OH) can be enhanced by loading iron phthalocyanine molecules onto them (FePc/CNT-R) through the axial coordination of functional groups to the Fe center atoms in iron phthalocyanine molecules (Fig. 4a). The FePc/CNT-NH_2_ with NH_2_ axial coordination exhibited an *E*_1/2_ of 0.92 V and remarkable electrocatalytic activity towards ORR (Fig. 4b). Cao et al. [110] reported a novel FePc-Py-CNTs electrocatalyst through covalent functionalization of single-walled CNTs with FePc. In this catalyst, pyridine was modified on CNT and coordinated to the Fe atom in FePc, forming a unique N-axial coordination structure (Fig. 4c). This structure exhibited superior electrocatalytic activity for ORR, with an *E*_1/2_ of 0.915 V compared to the benchmark Pt/C catalyst (*E*_1/2_ = 0.88 V), and demonstrated robust durability when cycled in an alkaline medium. To elucidate the origin of enhanced catalytic activity and excellent durability of FePc-Py-CNTs catalyst, researchers employed spin polarization DFT to calculate both FePc models with and without axial coordination. It was found that in comparison with the FePc-CNT system, the FePc-Py-CNT system exhibits a higher degree of O–O bond stretching, facilitating easier dissociation of O_2_ molecules. The binding energies of O_2_ and *OOH on FePc-Py-CNT are slightly higher than those of FePc-CNT, indicating a more favorable adsorption process on active sites in FePc-Py-CNT and hence superior electrocatalytic ORR performance. Moreover, Xia et al. [111] immobilized FePc onto N-doped graphene nanonets (NGMs), where the axial interaction between the Fe–N_4_ moiety of FePc and the N in the NGM graphene matrix generates an Fe–N_5_ structure exhibiting exceptional catalytic activity for ORR.

In addition to FePc, CoPc is also widely studied to be anchored on CNTs for ORR via the axial coordination with the functional groups on CNTs. Vier et al. [112] synthesized three distinct Co catalysts, namely CoPc, perfluoro CoPc (16(F)CoPc), and cobalt octaethylhexyl phthalocyanine (8(2-Et-C_6_H_11_O)CoPc), which were anchored on the pyridine (Py)-modified CNT. In these Co catalysts, the Co atom is coordinated with five N atoms, with an axial N from the Py. The electron paramagnetic resonance (EPR) and XPS spectra revealed that the signals of CNTs modified with various CoPcs exhibited distinct differences. Specifically, 8(2-Et-C_6_H_11_O) CoPcPy-CNT features a high-spin (*S* = 3/2) Co(II) species at its metal center, while both CoPc-Py-CNT and 16(F)CoPcPy-CNT comprise of low-spin configuration (*S* = 1/2) Co(II) species and intermediate spin (*S* = 1) Co(III) species, respectively. Further studies indicated that the pyridine ligand in 8(2-Et-C_6_H_11_O) CoPcPy-CNT and CoPc-Py-CNT functions as an electron-withdrawing group, while that in 16(F)CoPc-Py-CNT acts as an electron-donating group. This is attributed to the pull–push electronic effect induced by F residues within the coordinated Co–N_4_ atomic plane. But the impact of axial coordination surpasses that of the same-plane residues attached to the molecule. Therefore, the three complexes investigated in this study demonstrated comparable ORR activity, with a total electron transfer number of approximately 3.2 and a Tafel slope of around –50 mV dec^–1^.

Besides the N-containing functional groups or molecules on carbon-based substrates, other types of N-containing axial ligands have also been investigated to modify the structures and properties of metal phthalocyanines. Pizarro et al. [56] constructed four self-assembled catalysts with different configurations by binding pyridine salt molecules to FePc and then anchoring them on the surface of Au (111) electrodes. They investigated the effects of axial coordination of two pyridine isomers (Up and Down) with FePc and 16(Cl)FePc on the ORR properties of catalysts. DFT calculations showed that pyridine molecular wire, as an axial ligand, can decrease the electron density of the active site and alter Fe–O_2_ binding. Therefore, pyridine molecules play a crucial role in regulating and enhancing the activity of FePc towards ORR. After conducting electrochemical tests to study the ORR properties of various catalyst configurations, it was discovered that among all self-assembled catalysts tested, the Au (111)/Up/FePc system exhibited the highest level of catalytic activity.

DFT calculations also predict that the Fe–N_5_–C SACs with axial N coordination could exhibit superior electrocatalytic ORR activity compared to the typical Fe–N_4_–C SACs [113]. In addition to the Fe–N_5_ active sites engineered on FePc, other Fe–N_5_ SACs with axial N ligand have also been achieved, and demonstrate high electrocatalytic ORR activity. Liu et al. [114] successfully synthesized an Fe–N_5_/C@G SAC with an Fe–N_5_ active site on the surface of single-layer graphene utilizing FePc powder as a precursor. In the ORR process, an efficient 4e^–^ transfer process occurred on the Fe–N_5_/C@G catalyst. Lin et al. [115] have successfully synthesized Fe-SAC/N–C catalysts featuring axial N-coordination in Fe–N_5_ structure which exhibits excellent ORR activity (*E*_1/2_ = 0.89 V) and improved stability (Fig. 4d). Through DFT calculations, the ORR mechanisms of three SAC catalyst models (Fe–4pN, Fe–4pN–OH, and Fe–4pN–py) were studied and compared (Fig. 4e). It was discovered that axially coordinated pyridine can effectively regulate the interaction strength between Fe atoms and O-containing intermediates, thereby enhancing ORR activity. Li et al. [116] synthesized Fe–N–C/rGO-SAC, which features distinctive penta-coordinated Fe centers bound to five N atoms (Fig. 4f). The single Fe site is stabilized by four equatorial and one axial N atoms provided by a N-doped carbon matrix and an imidazole ring, respectively. This results in the formation of an asymmetric electron depletion region at the metal center, improving the electrocatalytic activity of ORR. It has also been reported that precise adjustment of the coordination number of Mn single atoms can significantly enhance ORR activity. Qin et al. [117] successfully synthesized atomically dispersed Mn–N_5_ catalyst, which exhibited lower energy barrier and higher O_2_ adsorption performance compared to traditional unit point Mn–N_4_ catalyst and Pt/C due to its uneven distribution of electronic charges. This accelerates ORR kinetics and results in a significant increase in catalyst activity.

#### Oxygen (O) Ligand Axially Coordinated SACs for ORR

Oxygen (O)-containing ligands (such as O, OH, etc.) are also an important class of axial coordination groups in modifying the M–N–C configuration. The electronic structure can be tuned by axial O-containing ligands, which in turn adjust the binding energy between intermediates and active sites. This adjustment effectively enhances the catalytic activity of a single metal atom. In recent years, studies on SACs with axial coordination of O-containing ligands have been developed, and various atomic configurations have been documented [118].

**Fig. 5:**
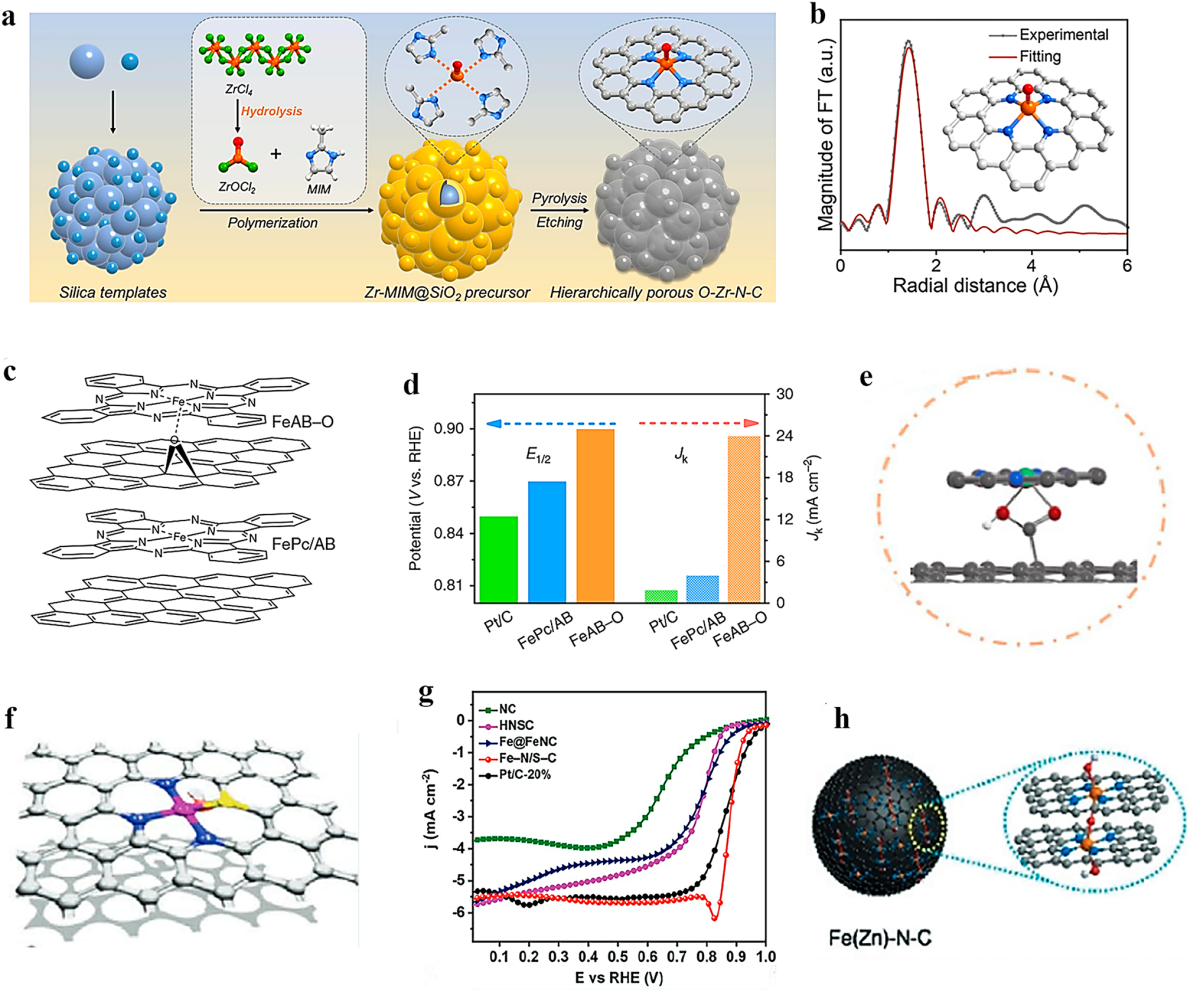
**a** Schematic diagram illustrating the synthetic route of O–Zr–N–C. **b** R space curve-fitting of O–Zr–N–C. Inset depicts the fitted structure of Zr site in O–Zr–N–C, the spheres in grey, blue, orange, and red represent C, N, Zr, and O atoms, respectively. Reproduced with permission [120]. Copyright: 2022 Wiley-VCH GmbH. **c** Molecular structure models of FeAB–O and FePc/AB. **d** *E*_1/2_ and *J*_k_ values at 0.88 V for FeAB-O, FePc/AB, and Pt/C. Reproduced with permission [124]. Copyright: 2020, Springer Nature. **e** Structural model of N_4_Ni_1_O_2_. Reproduced with permission [126]. Copyright: 2022 Wiley-VCH GmbH. f Structural model of Fe–N/S–C. g LSV polarization curves of NC, HNSC, Fe@FeNC, Fe–N/S–C and Pt/C at 1600 rpm in 0.1 M KOH aqueous solution. Reproduced with permission [127]. Copyright: 2021 Wiley-VCH GmbH. h The structural model of Fe(Zn)–N–C catalyst. Reproduced with permission [128]. Copyright: 2020 Wiley-VCH Verlag GmbH & Co. KGaA, Weinheim

The exploration of functional substrates and precise control over the electronic structure of atomic metal active species with a medium spin state holds great significance [119]. According to the transition metal *d-band* center theory, the performance of the catalyst is governed by the electronic structure of its catalytic center, which in turn determines the adsorption kinetics of intermediates. Wang et al. [120] reported Zr single atom site with a five-coordination configuration including an axial O ligand (denoted as O–Zr–N–C). The structure of O–Zr–N–C and its synthetic process are shown in Fig. 5a, b. The presence of the O axial ligand results in a lowered *d-band* center of Zr, which contributes to the stable local structure and appropriate adsorption capacity for intermediates. As a result, the ORR performance of O–Zr–N–C is significantly better than that of commercial Pt/C, with an *E*_1/2_ of 0.91 V and excellent durability, as demonstrated by a current retention rate of 92% after 130 h. Similarly, Zhang et al. [121] also synthesized Co-SAC with axial O coordination (Co-SA@N-CNFs). The local coordination configuration of single Co atom was proposed as a Co–N_4_O portion with an O atom in the axial direction perpendicular to the Co–N_4_ plane. Such axial coordination design contributed to excellent ORR activity. Compared to Pt/C (62 mV dec^–1^), the Co-SA@N-CNFs showed impressively low Tafel slope of 50 mV dec^–1^, highlighting its exceptional ORR kinetics.

In addition to the commonly reported plane-symmetric M–N_4_ configuration with axially coordinated O atoms, the asymmetric coordination structure of the central metal single atoms at the plane can also be further modified with additional axial O coordination. For example, the valence state of V element is variable, and its electronic structure can be modulated by an appropriate coordination structure. Cheng et al. [122] constructed a unique V–N_1_O_4_ co-coordinated with N and O embedded in a carbon matrix utilizing glycine as a chelating agent. In this compound, V is coordinated with one N and three O atoms to form a planar structure, while the other O atom is located axially at the center of the V atom. The V-SACs exhibited an *E*_1/2_ of 0.865 V in alkaline solution and demonstrated favorable performance even under acidic conditions. Likewise, Liu et al. [123] proposed an axial Fe–O–Ti ligand-regulated spin state transition strategy to enhance the performance of ORR. An axial Fe–O–Ti bridge bond was initially established in FeN_4_–O–Ti and FeN_3_O–O–Ti models, and the DFT calculations were conducted. The differential charge distribution of FeN_3_O–O–Ti is wider than that of FeN_4_–O–Ti, indicating a larger area of electrons participation in the electronic transition of FeN_3_O–O–Ti. As a result, the magnetic moment of Fe increases from 1.51 *μ*_B_ (FeN_3_O) to 3.52 *μ*_B_ (FeN_3_O–O–Ti), which facilitates favorable e–g filling and enhances the low-to-medium spin transition of Fe with increased O_2_ affinity. Moreover, compared to other configurations, FeN_3_O–O–Ti exhibited superior O_2_ adsorption energy (Δ*E*_ads_(O_2_), 1.84 eV) and integrated-crystal orbital Hamilton population (ICOHP) value (1.88 eV), resulting in its optimal binding strength with O_2_. It concludes that FeN_3_O–O–Ti could possess exceptional ORR activity. Based on this conclusion, a novel FeN_3_O–O–Ti catalyst named o-MQFe was synthesized and subjected to electrochemical performance testing. Consistent with the DFT calculation results, the synthesized catalyst demonstrated exceptional ORR activity, as evidenced by *E*_1/2_ and *E*_onset_ values of 0.861 and 0.96 V, respectively.

Axial O ligands are also used to modify the ORR properties of FePc. Chen et al. [124] proposed an electron localization strategy through axial Fe–O coordination to enhance O_2_ adsorption, and ultimately leads to high ORR performance. They designed a catalyst (FeAB–O) by coordinating FePc molecules with O-functionalized groups on the acetylene black (AB–O) treated by O_2_ plasma (Fig. 5c). The material exhibited exceptional ORR performance, with a remarkable *E*_1/2_ of 0.90 V and a calculated kinetic current density (*J*_k_) of 24.0 mA cm^−2^ at 0.88 V, surpassing FePc/AB (*E*_1/2_ = 0.87 V, *J*_k_ = 1.9 mA cm^−2^ at 0.88 V) and Pt/C (*E*_1/2_ = 0.85 V, *J*_k_ = 4.0 mA cm^−2^ at 0.88 V) (Fig. 5d). Xu et al. [62] reported a Fe@Fe/N-G-800 SAC material, which was designed and synthesized by bonding FePc molecules to graphene-like Fe–N–C materials with axial O–FeN_4_ coordination sites. The central Fe atom in FePc can coordinate with the O atom in O–FeN_4_ site from Fe/N-G-800, thus forming a unique Fe–O–Fe structure. The formation of Fe–O–Fe bridge bonds between Fe–N_4_ sites effectively reduced ORR overpotential. While axial coordination between metal atoms and O atoms has significantly increased the ORR electrocatalytic activity of most SACs, it cannot be simply concluded that such coordination inevitably leads to improved electrocatalytic ORR activity. Cao et al. [125] prepared Co-SACs by coordinating CoPc with O atoms from different positions in the carbon plane and evaluated their electrochemical ORR performance. They found that the ORR electrocatalytic performance of the catalyst could be significantly enhanced only when the axial O was located on the carbon defect sites.

In addition to a single axial O atom ligand, some other types of axial O ligands have also been investigated and documented, including those with two O atoms or OH groups. Xiao et al. [126] synthesized Ni-SAC with Ni atom coordinated by four planar N atoms and two axial O atoms (N_4_–Ni–O_2_) loaded on carboxyl functionalized MCNTs, as illustrated in Fig. 5e. In contrast to the other type Ni-SACs synthesized, the N_4_–Ni–O_2_ SAC exhibited a greater propensity towards 2e^−^ ORR, with a H_2_O_2_ Faradaic efficiency (FE) of about 96% at a current density of 200 mA cm^−2^, surpassing other reported SACs. This research is one of the few that aim to enhance the performance of 2e^−^ ORR through axial O ligand modification of SACs. Similarly, Xiao et al. [59] employed the zeolite imidazolium framework (ZIF-8) as a self-template to synthesize Fe-SACs with uniform dispersion, in which each Fe atom coordinates with four N atoms in the plane and two O atoms in the axial direction. The optimized Fe–N–C catalyst demonstrates outstanding ORR activity in both acidic and alkaline solutions, with *E*_1/2_ of 0.81 and 0.90 V, respectively. This multiple axial coordination design offers a new strategy for further optimizing SACs for various reactions. Li et al. [127] prepared Fe–N/S–C catalysts with axial OH coordination by embedding an asymmetric N–S coordinated Fe single atom into N–S co-doped porous carbon nanospheres (Fig. 5f). The quantitative structural parameters of Fe in Fe–N/S–C were obtained by EXAFS fitting, which revealed that the coordination numbers for the Fe–N, Fe–S, and Fe–O bonds were 2.8, 1.2, and 1.0, respectively, forming an Fe–N_3_S_1_–OH structure. The prepared catalyst exhibited exceptional electrocatalytic activity for ORR ascribing from its high specific surface area, open-layered porous structure, and excellent conductivity. Among all tested samples, Fe–N/S–C showed the highest *E*_onest_ of 0.970 V and *E*_1/2_ of 0.882 V. Additionally, this catalyst demonstrated remarkable methanol resistance and durability (Fig. 5g). Gong et al. [128] proposed a novel selective manipulation strategy aimed at introducing axial O to the Fe site, which led to the successful preparation of Fe(Zn)–N–C catalysts. Interestingly, in this catalyst, O and OH are co-axially coordinated at the central site of the single atom of Fe. The O modification is stabilized by forming an axial Fe–O–Fe bridge bond, while the remaining axial coordination positions at the two FeN_4_ sites are connected to OH groups (Fig. 5h). The resulting modulation of energy levels confers these sites with intrinsic activity more than 10 times higher than that of ordinary FeN_4_ sites, thereby enabling the development of ORR electrocatalysts with significantly enhanced activity.

#### Sulfur (S) Ligand Axially Coordinated SACs for ORR

**Fig. 6:**
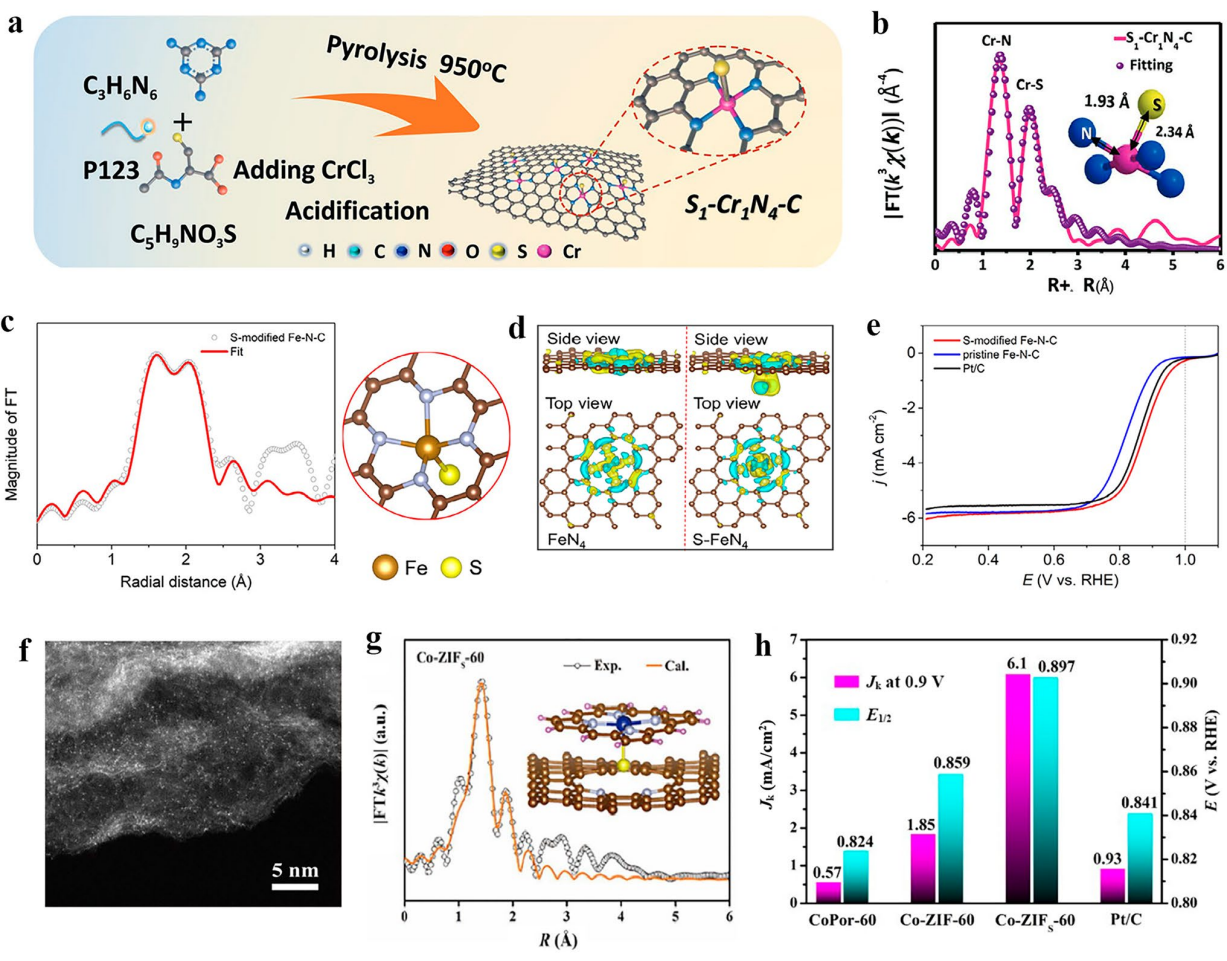
**a** Synthetic procedure of the Cr single-atom anchored on N, S co-doped porous carbon nanosheet (S_1_–Cr_1_N_4_–C). **b** Cr K-edge EXAFS fitting analysis of S_1_–Cr_1_N_4_–C in K space. Reproduced with permission [129]. Copyright: 2023 Wiley-VCH GmbH. **c** EXAFS fitting at R space for S-modified Fe–N–C and the proposed model for S–FeN4. **d** Spatial charge density difference isosurfaces of S–FeN4 and FeN4. Yellow and blue iso surfaces represent electron accumulation and electron depletion, respectively. **e** LSV curves at 1600 rpm of S-modified Fe–N–C, pristine Fe–N–C, and Pt/C. Reproduced with permission [130]. Copyright: 2023 Wiley-VCH GmbH. **f** AC-HAADF-STEM image of Co-ZIFS-60 sample. **g** The experimental and calculated FT-EXAFS spectra of Co1N4-thiophene S1 (Co-ZIFS-60) based on the DFT model shown in the inset. Schematic model, Co (blue), N (blue), C (gray), S (yellow), and H (pink). **h** Comparison of *J*_k_ at 0.90 V and *E*_1/2_ of different samples. Reproduced with permission [131]. Copyright: 2022, Springer Nature Switzerland AG. (Color figure online)

In recent years, researchers have successively reported M–N_4_ SACs with different metal atomic centers that coordinate axially with S ligands. The studies have confirmed that axial S-ligands can disrupt the electronic localization around the planar M–N_4_ active center, thereby promoting the rate-limited reduction release of *OH and accelerating the entire ORR process. Using the synthetic route shown in Fig. 6a, Guo et al. [129] have successfully created an atomically dispersed Cr catalyst featuring a five-coordinated active site (S_1_–Cr_1_N_4_–C). Through various characterization methods, including an EXAFS study, it was determined that S_1_–Cr_1_N_4_ comprises of four Cr–N bonds, each with a length of 1.34 Å, and one Cr–S bond with a length of 2 Å. It was thus inferred that the configuration of single Cr atom site is most likely an axially S-coordinated Cr_1_N_4_ configuration (Fig. 6b). The resulting S_1_–Cr_1_N_4_–C catalyst exhibited significantly improved ORR activity, enhanced methanol tolerance, and superior stability. In similar research, Li et al. [130] synthesized a controllable local coordination environment for an S-modified Fe–N–C catalyst, where the Fe atom was coordinated with four in-plane N atoms and one external axial S atom (as depicted in Fig. 6c). DFT calculations indicated that the electron density of the Fe atom in S–FeN_4_ is lower than that of the Fe atom in FeN_4_ (Fig. 6d). This observation suggests that the presence of the external S atom influences the electronic distribution of the active site of FeN_4_ as well as the spin state of Fe. The emergence of a higher valence state and spin state of Fe signifies an augmentation in the number of unpaired electrons. As a result, an optimized reactant adsorption and desorption capability was achieved at the active site of FeN_4_, leading to enhanced ORR activity with the most positive *E*_onset_ of 0.99 V and *E*_1/2_ of 0.88 V among all catalysts (Fig. 6e). Moreover, Chen et al. [131] developed a strategy that combines matrix activation with controlled induction to design and construct Co_1_N_4_–S_1_ active site with axial Co–S coordination by inducing the coordination of cobalt Por (CoPor) molecules with S and N co-doped carbon materials. The AC-HAADF-STEM image and corresponding structure details of the active site are illustrated in Fig. 6f, g. Benefiting from these unique structural characteristics, Co_1_N_4_–S_1_ showed high ORR reactivity and remarkable ORR kinetics in alkaline solution, with *E*_1/2_ of 0.897 V and *J*_k_ of 6.1 mA cm^−2^ (Fig. 6h).

#### Halogen (Cl/I) Ligand Axially Coordinated SACs for ORR

**Fig. 7:**
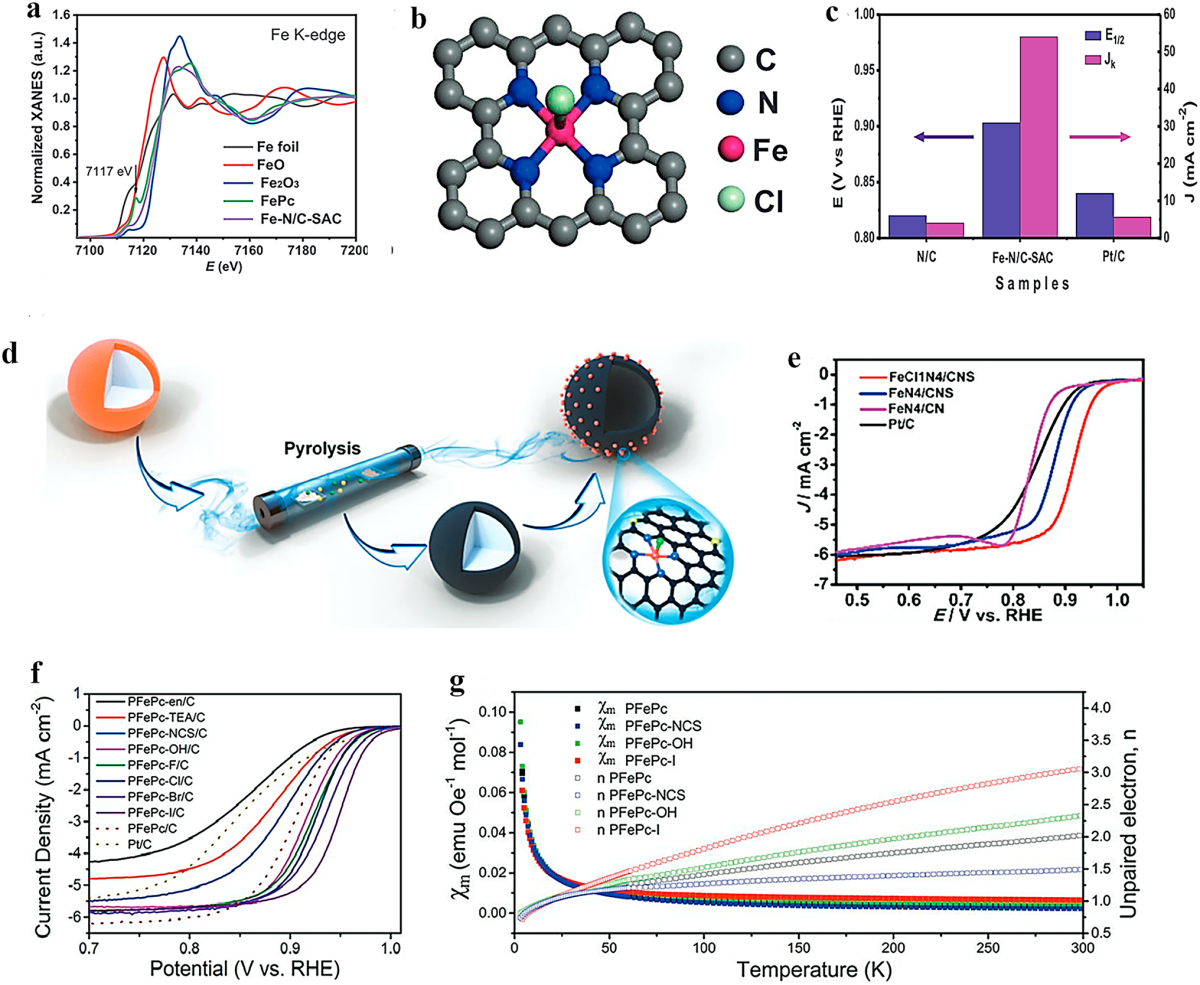
**a** Fe K-edge XANES spectra for Fe–N/C-SAC. **b** The proposed Cl–Fe–N_4_ structural model. Fe, N, Cl, and C atoms are shown in pink, blue, green, and gray, respectively. **c** Comparison of *J*_k_ at 0.85 V and *E*_1/2_ of N/C, Pt/C, and Fe–N/C-SAC. Reproduced with permission [132]. Copyright: 2021 Wiley-VCH GmbH. **d** Schematic illustration of the synthesis of FeCl1N4/CNS. **e** ORR polarization curves in O2-saturated 0.1 M KOH. Reproduced with permission [133]. Copyright: 2018 The Royal Society of Chemistry. **f** LSV curves at a scan rate of 10 mV s^−1^ on the as-prepared PFePc-L/C and Pt/C electrodes under a rotation rate of 1600 rpm in a 0.1 M KOH electrolyte at room temperature. **g** M–T curves and the calculated number of the unpaired electrons in the Fe 3d orbitals in PFePc, PFePc–NCS, PFePc–OH, and PFePc–I. Reproduced with permission [138]. Copyright: 2022 Wiley-VCH GmbH. (Color figure online)

Apart from typical axial ligands that contain N, O and S atoms, halogen ligands can also be introduced to axial coordination site of M–N–C catalysts. Axial Cl coordination offers an effective means for modulating the surface electronic structures, thereby expediting the 4e^−^ pathway kinetics by near-optimal adsorption of intermediates. Xin et al. [132] synthesized Fe–N/C SACs, and the structural characteristics of the catalyst were confirmed by EXAFS analysis. The Fe atom is situated at the center of a double-vacancy cavity, which is bonded by four in-plane N atoms and one axial Cl atom, forming the FeN_4_–Cl active site anchored within the graded porous carbon matrix (Fig. 7a, b). The catalytic performance of Fe–N/C SACs is enhanced by the Cl axial coordination to the active site, exhibiting excellent alkaline ORR activity with *E*_1/2_ = 0.91 V and *J*_k_ up to 55 mA cm^−2^ at 0.85 V in 0.1 M KOH, which are respectively 20.8 and 11.5 times higher than those of N/C and Pt/C catalysts (Fig. 7c).

Notably, the ORR catalytic activity of SAC can be further increased through the synergistic effect of axial Cl coordination and S-doping in the carbon substrate. Using the thermal migration method, Han et al. [133] fabricated an atomically dispersed FeCl_1_N_4_/CNS catalyst. Figure 7d, e illustrates respectively the synthetic strategy of FeCl_1_N_4_/CNS and the outstanding ORR activity of the catalyst in alkaline solution, with *E*_1/2_ = 0.921 V, surpassing that of non-noble metal electrocatalysts reported to date, including FeN_4_/CN counterpart. Experiments and DFT calculations demonstrated that the electronic state of a single Fe active center can be modulated by its surrounding chemical environment, elucidating the reason for the superior ORR activity of FeCl_1_N_4_/CNS. Besides, Zhang et al. [134] obtained axial Cl coordinated Fe–N_4_ catalyst bearing resemblance to tetraphenyl porphyrin (FeTPPCl) with the FeN_4_ component predominantly adopting a twisted square-cone coordination geometry. The synergy between the rich N-doping in the carbon substrate and the axial Cl coordination in Fe–N_4_ active site enables the catalysts an outstanding ORR performance.

To uncover the origin of the high ORR activity after axial coordination, Ding et al. [135] synthesized Cl-axially modified Fe–N–C SACs (FeN_4_Cl SAC) to evaluate its electrocatalytic performance for ORR. Subsequently, they constructed various structural models for FeN_4_Cl SAC and conducted DFT calculations to further clarify the nature of active sites. The results showed that the original FeN_4_Cl–C configuration exhibited the most significant exothermic behavior and strongest ORR activity compared to FeN_4_Cl (FeN_4_Cl–C-1 and FeN_4_Cl–C-4) with carbon defect structures. The introduction of Cl facilitated charge transfer within the Fe active site from N atom to Cl atom in the graphene structure, thereby enhancing the ORR activity. The projected density of states (PDOS) of different configurations of Fe d-orbitals, N p-orbitals, and Cl p-orbitals further demonstrates the exceptional ORR catalytic activity of FeN_4_Cl–C. Compared to FeN_4_–C SAC, the FeN_4_Cl–C configuration exhibits a stronger intensity near the Fermi level, which can lead to an enhancement in ORR activity.

In recent years, the utilization of metal–organic frameworks (MOFs) as precursors for the preparation of SACs has attracted significant attention from researchers [136]. As a type of porous crystalline solids, MOFs with readily structural modifications hold great potential as precursors/self-sacrificial templates for the synthesis of SACs with axial coordination. Hu et al. [64] obtained SACs by pyrolysis of 4,5-dichloro imidazole-modified Zn/Fe- bimetallic triazole (MET) framework with high N content. Thanks to the unique properties of MET with rich concentration of N atoms in triazole, the high-density Fe singe-atom with an FeN_4_Cl_1_ configuration was achieved. FeN_4_Cl_1_/NC shows excellent ORR activity in both alkaline and acidic electrolytes. DFT calculations demonstrate that the presence of Cl can optimize the adsorption free energy of Fe sites for *OH, thus promoting the ORR process. Besides the commonly used ORR active transition metals like Fe, the generally non-active transition metal elements can also exhibit high ORR activity by axial coordination design in their corresponding SACs. For example, Ji et al. [137] synthesized various YN_4_ SACs with different axial coordination designs using different ligands. After comparing a series of axial ligands, it was discovered that the axial Cl coordination can significantly enhance the ORR activity of YN_4_ SAC in alkaline solutions, which is comparable to that of Pt/C catalyst. DFT calculations found that there was moderate coupling between the 3p orbital of Cl atom and the 4d orbital of Y, so the covalent bond of YN_4_–Cl was extended adaptively to promote the binding of intermediates in ORR process.

In addition to Cl ligand, I ligand has also been utilized in the investigation of the axial coordination of SACs. To elucidate the actual structure and catalytic mechanism of Fe–N_4_ SAC, Zhao et al. [138] used poly (iron phthalocyanine) (PFePc) as a model electrocatalyst and investigated the ORR catalytic activity mechanism via axial coordination control on the active site. They selected a series of ligands with different field strengths including strong-field ligands such as ethylenediamine (en), triethylamine (TEA), and NCS, and weak-field ligands such as OH, F, Cl, Br, I to axially coordinate with the Fe–N_4_ site in PFePc (denoted as PFePc–L, L = axial ligand). The study revealed a positive correlation between ligand field strength and the ORR catalytic activity of Fe–N4, as evidenced by the gradual increase in both *E*_1/2_ and *J*_k_ of ORR on the PFePc-L/C electrode with decreasing ligand field strength (Fig. 7f). In particular, the PFePc–I/C catalyst displayed a remarkably high *E*_1/2_ of 0.948 V, which stands out as the highest among all reported Fe–N_4_ based catalysts. Experimental results indicated that by axially coordinating the Fe center with ligands of varying field strengths, the three-dimensional orbital configuration and electron spin state of Fe–N_4_ can be manipulated according to the crystal-field theory (Fig. 7g). DFT calculations further demonstrated that axial ligand coordination not only rearranges the Fe 3d-orbital configuration but also leads to a reduction in orbital energy levels, resulting in higher ORR activity.

#### Other Ligands Axially Coordinated SACs for ORR

Apart from the aforementioned heteroatom-based axial ligands, carbon-based ligands can also function as axial coordination ligands in SACs. In such cases, the axial C atom is usually from the carbon support, this coordination aims to enhance the metal-support interaction and stabilize the metal atom in the catalyst. In the study conducted by Yan et al. [139], the FePc molecules were initially subjected to oxidation and doping via the Hummer method. Then, carbon-anchored FePc composites (FePc-RCNTs) were synthesized by integrating FePc precursor and oxidized MWCNTs at room temperature, in which an axial covalent bond between the carbon matrix and Fe–N_4_ site was established. FePc-RCNTs exhibited a positive *E*_1/2_ of 0.86 V in ORR, and the Tafel analysis indicated that the strong interaction between FePc and MWCNTs facilitated the kinetic process in ORR catalysis.

In addition to direct coordination with C atoms from carbon substrate, other C-containing groups as axial ligands are also reported. For example, Luo et al. [140] designed a two-dimensional metal–organic material Fe-Pp flake with an axial cyanide (–CN) ligand. DFT calculation revealed that the Fe-Pp–CN sheet exhibits higher ΔG_*OOH_, ΔG_*O_, and ΔG_*OH_ values compared to those of the pristine Fe-Pp sheet, indicating a weakened binding interaction between these intermediates and the Fe-Pp sheet upon axial CN ligand coordination. Combined with external tensile strain, the ORR activity of the Fe-Pp–CN sheet can be further enhanced, even surpassing that of Pt. In order to evaluate the impact of axial C ligands, researchers employed DFT calculations to conduct a series of investigations into the effects of various axial ligands on the catalytic performance of SACs for ORR. For instance, She et al. [141] studied the ORR performance of CrN4-Gra modified with diverse axial C ligands, including –CH, –CH_2_, –CH_3_, –NH, –NH_2_, –NH_3_, –C_6_H_5_ (benzene), –C_6_H_5_–NH_2_ (aniline), and –C_6_H_5_–NO_2_ (nitrobenzene). It was determined that CrN_4_-Gra modified with ligands –CH_3_, –C_6_H_5_, –C_6_H_5_–NH_2_, and –C_6_H_5_–NO_2_ exhibited favorable ORR activity with low overpotentials of 0.37, 0.35, 0.37, and 0.29 V respectively. Moreover, after axial coordination of these ligands with various MN_4_-Gra compounds, they further found that –C_6_H_5_–NO_2_ and –C_6_H_5_ exhibit the highest catalytic activity. Specifically, FeN_4_-Gra/C_6_H_5_–NO_2_ demonstrated the highest catalytic activity, followed by RuN_4_-Gra/C_6_H_5_–NO_2_ and FeN4-Gra/C_6_H_5_. These findings provide valuable guidance for future experimental investigations into highly efficient ORR catalysts. Furthermore Lu et al. [142] conducted a systematic investigation on the catalytic activity and ligand coordination effects of 17 five-coordinated Fe–N–C catalysts (Fe–N–C–X, X represents axial ligands) through DFT calculations. The findings indicated that the axial coordination effect can diminish the orbital hybridization between Fe active sites and ORR-related intermediates, thereby expediting ORR. More importantly, it was observed that the catalytic activity of Fe–N–C–X exhibited an upward trend as the electronegativity of the X ligand decreased. Among the 17 Fe–N–C catalysts modified with axial ligands, the –SCN ligands modified electrocatalyst exhibited the most favorable OH adsorption energy, resulting in superior ORR activity and a lower overpotential of 0.28 V.

Phosphorus (P) is another heteroatom-based axial ligand reported for the modification of SACs. Studies have shown that the Co single atom coordinated by N and P-doped porous carbon (Co–N, P–C) exhibits slightly higher ORR catalytic activity compared to that coordinated by only N-doped porous carbon (Co–N–C), indicating the advantageous role of P-related species in ORR electrocatalysis [143]. Therefore, employing P-related species as axial ligand in SACs has emerged as a promising strategy to enhance their ORR performance. Zhu et al. [144] synthesized a hollow carbon structure embedded with N, P-coordinated iron atoms (FeNPC) via a facile polymerization-carbonization route. The co-coordination of Fe atoms with N and P on the surface of carbon spheres serves as the active center for ORR, where FeN_4_ is axially coordinated with PO_4_ group. Impressively, the prepared FeNPC catalyst showed remarkable ORR performance in both alkaline and acidic electrolytes.

Besides the well-documented non-metallic heteroatoms serving as axial ligands, principally single metal atoms, metalloid clusters and metal-containing molecules may also function as ligands to fine-tune the ORR electrocatalytic activity of SACs. However, research on the axial coordination of SACs with metal ligands is limited. Zeng et al. [145] synthesized Pt single-atom grafted Fe–N–C SACs (Pt_1_@Fe–N–C), which introduced a novel active site of Pt_1_O_2_–Fe_1_N_4_ with axial coordination of Pt_1_O_2_. They employed X-ray absorption spectroscopy, encompassing both XANES and EXAFS techniques, to propose two plausible configurations for the novel Pt_1_O_2_–Fe_1_N_4_ moiety in Pt_1_@Fe–N–C. In PEMFC testing, the grafted Pt_1_O_2_ not only imparted Pt_1_@Fe–N–C a high power density, but also had a protective effect on slightly reduced Fe^3+^ atoms, thereby mitigating the catalytic Fenton's reaction of Fe centers. This approach presents a novel perspective on the Fe–N–C system and its potential functional expansion. In addition to metal atoms, metalloid clusters can also act as axial ligands in SACs. By mimicking the conformational kinetics of enzymes during the reaction process, Ji et al. [146] introduced p-block metalloid cluster Te_n_ into the pyrolytic FeN_4_-carbon framework to synthesize an axial Te_n_ ligand modified catalyst (FeN_4_–Te_n_). The atomic-resolution aberration-corrected HAADF-STEM revealed the well-dispersed Fe atoms in the carbon support, and a significant number of Te clusters were present in the catalyst. Fe single atoms (Fe-SAs) were observed to be distributed around Te clusters, indicating a strong interaction between them. Synchrotron radiation XAS measurements at the Fe K-edge further determined the chemical state and coordination environment of FeN_4_–Te_n_ at the atomic level. The results indicate that the isolated Fe single-atom is coordinated by four N atoms and approximately one Te atom. Further investigation demonstrated that the FeN_4_–Te_n_ exhibited exceptional electrocatalytic ORR performance in alkaline medium. DFT calculations revealed that the coordination environment and electronic structure of the Fe center can be dynamically controlled by p–d orbital coupling between Te_n_ clusters and FeN_4_ elements when n > 2. Moreover, the presence of a fifth electron-absorbing Te_n_ cluster ligand results in additional weakening of the binding energy of *OH on Fe centers.

### Carbon Dioxide Reduction Reaction

The electrochemical reduction of CO_2_ into high-value chemicals and fuels is a promising avenue for utilizing renewable electricity and mitigating CO_2_ emissions [147], which has become a cutting-edge field of energy conversion and carbon neutrality [148]. Electrocatalytic CO_2_RR is a complex process involving multiple proton-coupled and electron-transfer steps. By adjusting different catalytic systems and electrode potentials, the selectivity of products can be regulated while optimizing the reaction rate under ambient temperature and pressure conditions. In the process of electrocatalytic CO_2_RR, CO_2_ can be reduced by 1, 2, 4, 6, and 8 electrons under the action of different catalytic systems. Reduction products can be broadly classified into two categories: C_1_ products (such as CO, formic acid, and methane) and C_2_/C_2+_ products (including ethylene and acetone). Due to the similar reaction potentials, HER is the main competitive reaction in the CO_2_RR process. According to the analysis of reaction pathways based on existing literature, it has been found that different products may share common initial or intermediate stages. Among various reaction intermediates, *CO plays a crucial role in both C_1_ and C_2_/C_2+_ reaction pathways. Based on some mainstream views, the *CO intermediates may undergo direct desorption, further hydrogenation to form C_1_ product, or experience dimerization and hydrogenation during C_2_/C_2+_ reaction pathway. This is closely related to the binding energies between intermediates and the surface of the catalysts.

In recent years, the development of SACs for CO_2_ conversion has rapidly progressed due to their high atomic utilization and demonstrated high specific product selectivity. SACs for CO_2_RR can be classified into two categories based on the type of active metal center: noble metals and non-noble metals. However, non-noble metal SACs are currently limited by their impractical current density and suboptimal catalytic selectivity. To meet the economic demands of large-scale industrial applications, it is critical to design and develop exceptional non-noble metal SACs with significant electrocatalytic performance. Various strategies have been developed to improve the electrocatalytic CO_2_RR performance of non-noble metal SACs, including coordination structure control [149], deposition of single atom sites onto highly porous supports [150], heteroatom doping, single atom alloying, increasing site density and introducing defect sites [151]. It is worth mentioning that the axial coordination of SACs has emerged to be an intriguing method for effectively regulating the catalytic activity, selectivity, and stability of single atom sites toward CO_2_RR. The introduction of axial ligands can lead to the formation of asymmetric coordination for metal single atoms, which disrupts the electronic structure balance of metal single atoms, resulting in variations in electron density distribution that ultimately impact catalyst activity. While classic SACs with M–N_4_–C planar structures exhibit excellent performance in electrocatalytic CO_2_RR, the high electronic structure symmetry of its M–N_4_ site impedes electron transfer during catalysis, which hinders the optimization of its catalytic performance. Therefore, designing axial coordination environment to break the electronic structure symmetry of M–N_4_ sites could be a feasible strategy for enhancing the intrinsic activity of central metal atoms and significantly improving the catalytic performance. As summarized in Table 2, the attempts reported so far mainly focus on M-N_4_ (M: Fe, Co, Ni), and the central transition metal atoms determine these catalysts tend to the CO_2_-to-CO reaction pathway, which has been proven in many previous studies. The axial coordination strategy can further suppress the side reaction HER and improve catalyst performance while keeping the main products unchanged. Therefore, our subsequent discussion will mainly focus on the impact of introducing axial coordination to SACs on electrocatalytic CO_2_RR for CO production.

**Table 2:** Summarized electrocatalytic CO_2_RR activity of typical axial coordination modified SACs

Axial-coordinated SACs	Coordination structures	Axial atom	Main products	FE^*a*^	Partial current density^*b*^	Stability (h)	Refs
Fe–N/CNT	Fe–N_5_	N	CO	95.47%@-0.6 V	–	10	[152]
Fe–SA/ZIF	Fe–N_5_	N	CO	98.0% @-0.7 V	7.1 mA cm^−2^@-0.7 V	40	[153]
Fe–N_5_/DPCF	Fe–N_5_	N	CO	93.1% @-0.5 V	9.4 mA cm^−2^@-0.49 V	25	[154]
Ni–N_5_–C	Ni–N_5_	N	CO	99.6% @-2.4 V	1.23 A cm^−2^@-2.4 V	100	[155]
FeN_5_	Fe–N_5_	N	CO	97.0%@-0.35 V	0.5 mA cm^−2^@-0.35 V	24	[156]
CoPc–py–CNT	Co–N_4_–py	N	CO	98.8%@-0.63 V	9.9 mA cm^−2^@-0.73 V	12	[157]
Co–N_5_/HNPCSs	Co–N_5_	N	CO	99.2%@-0.73 V	4.4 mA cm^−2^@-0.73 V	10	[158]
Ni SAs/OMMNC	Ni–N_4_–O	O	CO	99.0% @-0.6 V	5.1 mA cm^−2^@-0.6 V	17	[159]
NiSA–N–PGC	Ni–N_4_–O	O	CO	97.2%@-0.76 V	53 mA cm^−2^@-1.1 V	40	[160]
NiN_4_–O_2_–FePc	Ni–N_4_–O_2_	O	CO	97.65%@-0.5 V	252 mA cm^−2^@-0.5 V	20	[161]
Ni-NUK-900	Ni–N_4_–O	O	CO	94.0%@-0.73 V	3.4 mA cm^−2^@-0.73 V	12	[162]
O–Fe–N–C	Fe–N_4_–O	O	CO	95.0% @-0.5 V	4.4 mA cm^−2^@-0.5 V	30	[163]
Fe–N/O–C (MZ)	Fe–N_4_–O	O	CO	96.0%@-0.57 V	5.4 mA cm^−2^@-0.57 V	22	[164]
Fe–CON_400_-400	Fe–N_4_–O	O	CO	∼100%@-0.83 V	-	12	[165]
SnPc/CNT–OH	Sn–N_4_–O	O	HCOOH	89.4% @-1.0 V	74.8 mA cm^−2^@-1.0 V	8	[166]
Ni–N_4_–O/C	Ni–N_4_–O	O	CO	99.2% @-0.9 V	23 mA cm^−2^@-0.9 V	20	[66]
CdN_4_S_1_/CN	Cd–N_4_–S	S	CO	99.7%@-2.4 V vs Ag/Ag^+^	–	24	[167]
ZnN_4_S_1_/P–HC	Zn–N_4_–S	S	CO	~ 100% @-0.6 V	15.8 mA cm^−2^@-0.8 V	30	[168]
FeN4Cl/NC-7.5	Fe–N4–Cl	Cl	CO	90.5% @-0.6 V	10 mA cm-2@-0.8 V	15	[169]
(Cl, N)–Mn/G	Mn–N_5_–Cl	Cl	CO	97% @-0.6 V	14.3 mA cm^−2^@-0.8 V	–	[170]
Ni1–N–C(Cl)	Ni–N_4_–Cl	Cl	CO	94.7% @-0.7 V	2.75 mA cm^−2^@-0.7 V	10	[171]
Ni1–N–C(Br)	Ni–N_4_–Br	Br	CO	83.3% @-0.65 V	0.9 mA cm^−2^@-0.65 V	–	[171]
Ni1–N–C(I)	Ni–N_4_–I	I	CO	69.4% @-0.65 V	0.4 mA cm^−2^@-0.65 V	–	[171]

^a,b^The reported potentials are with respect to the reversible hydrogen electrode (V vs. RHE) unless otherwise stated

#### Nitrogen (N) Ligand Axially Coordinated SACs for CO_2_RR

Atomically dispersed Fe atoms immobilized on an N-doped carbon matrix have gained significant attention in the field of CO_2_RR. In most reports, Fe–N_4_ has been identified as the active site of Fe–N–C catalysts owing to their excellent CO_2_RR performance [172]. Based on the planar Fe–N_4_ structure, introducing an axial Fe–N bond to form an Fe–N_5_ structure is a common strategy for regulating the local coordination environment of the Fe site and enhancing CO_2_RR performance. By incorporating aminated CNTs, Tuo et al. [152] successfully fabricated an axial Fe–N_5_/CNT catalyst with four planar Fe–N coordination and one axial Fe–N coordination. Compared to the planar Fe–N_4_/CNT, the additional axial Fe–N coordination effectively lowered the energy barrier for CO desorption, suppressed HER occurrence, and enhanced CO selectivity during CO_2_RR process. Cheng et al. [153] synthesized an Fe–N_5_ single-atom catalyst (Fe-SA/ZIF) via pyrolysis, and demonstrated its potential as a highly efficient catalyst material. This catalyst exhibited an FE_CO_ of 98% at –0.7 V vs. RHE, surpassing the majority of reported SACs to date. Its superior performance can be attributed to the out-of-plane coordinated axial pyridinic N, which led to a negative shift of the *d-band* center and weakened the binding strength between the adsorbent and active site according to the *d-band* theory. The partial density of states (PDOS) calculation indicated that the *d-band* centers of FeN_4_ and FeN_5_ experienced varying degrees of negative shifts due to orbital hybridization between the C-2p orbit of the CO intermediate and Fe-3d orbit. The corresponding values are 0.91 and 1.15 eV for Fe–N_4_ and Fe–N_5_, respectively. Therefore, Fe–N_5_ with axial pyridine N coordination exhibited a greater advantage in CO desorption.

**Fig. 8:**
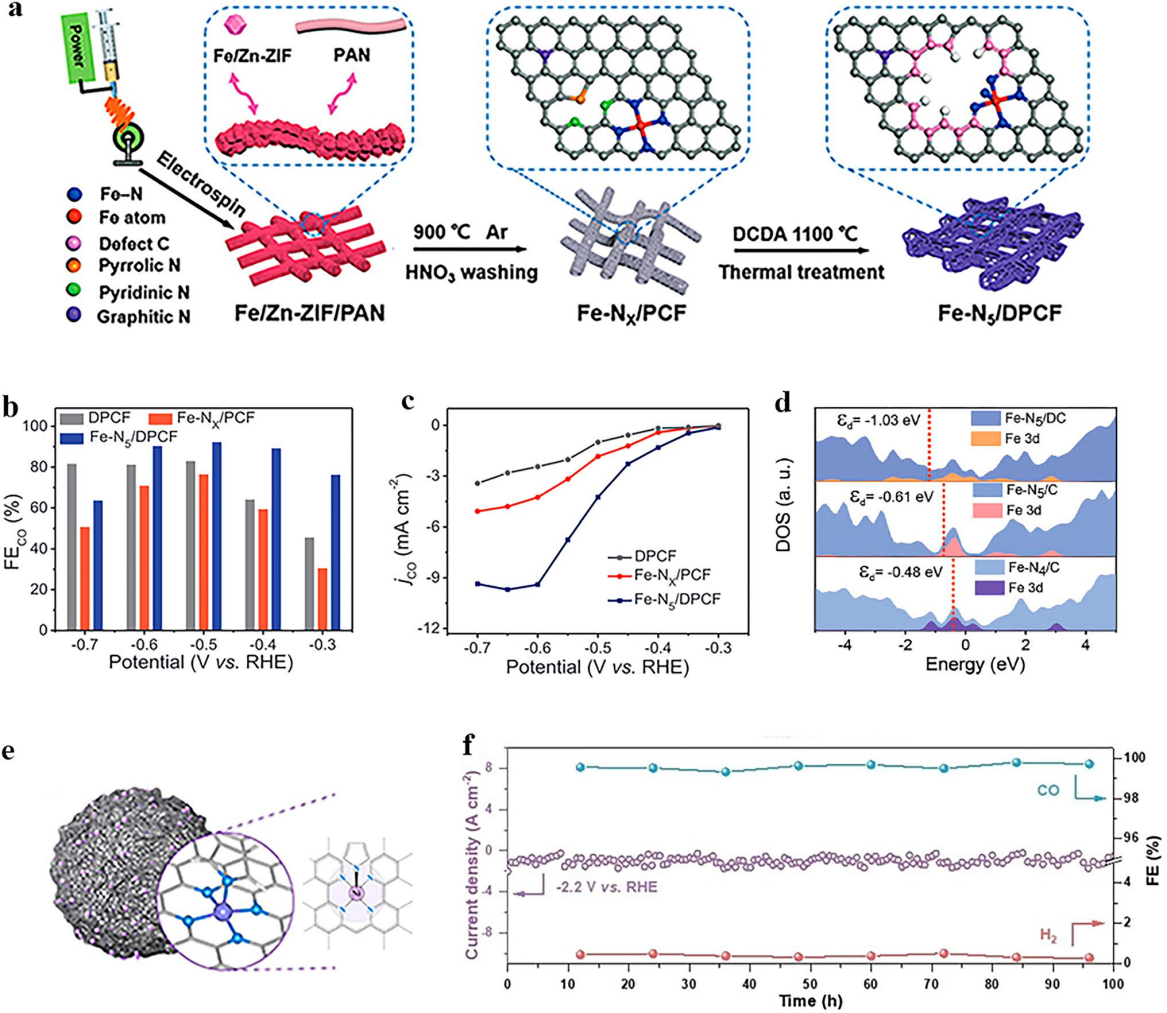
**a** Schematic diagram of the preparation procedure of Fe–N_5_/DPCF. **b** CO Faradaic efficiencies, and **c** CO partial current densities of DPCF, Fe–Nx/PCF, and Fe–N_5_/DPCF at different potentials. **d** The total DOS (red-shaded areas) and d-states of Fe atom (blue-shaded areas) in the optimized structures of FeN_4_/C, FeN_5_/C, and FeN5/DC. Reproduced with permission [154]. Copyright: 2022 Wiley-VCH GmbH. **e** Coordination configuration of the Ni–N_5_–C single-atom nanoenzyme. **f** Long-term stability test of Ni–N_5_–C for CO_2_RR operated at − 2.2 V vs. RHE. Reproduced with permission [155]. Copyright: 2022 Wiley-VCH GmbH

In addition, axial ligands can synergistically collaborate with defects to further improve the CO_2_RR performance of the catalyst. Li et al. [154] designed a facile electrospinning and two-step annealing strategy to successfully prepare Fe–N_5_ SACs on defect-rich porous carbon nanofibers, denoted as Fe–N_5_/DPCF (Fig. 8a). As shown in Fig. 8b, c, Fe–N_5_/DPCF exhibited significantly enhanced CO_2_RR performance across a broad potential range compared to Fe–N_x_/PCF. DFT calculations were performed to further investigate the structure–property–performance relationships resulting from axial coordination and defect-rich supports. The DOS calculation showed that the introduction of an axial N-ligand results in a negative shift in the *d-band* center of Fe atoms. Furthermore, due to the alterations in the nature of active site, defects further enhance this negative shift (Fig. 8d). As a result, the adsorption behavior of reactants and intermediates underwent changes during the reaction process, resulting in promoted *CO desorption and *COOH formation while inhibiting *H formation. Benefiting from these property modifications, the selectivity of the catalyst has been improved.

In addition to Fe–N–C materials, other M–N–C materials also show great potential as catalysts for CO_2_RR, but their current density and durability are still insufficient. To address this issue, researchers have made diverse endeavors. Huang et al. [155] synthesized a Ni–N_5_–C catalyst with an enzyme-like structure (Fig. 8e), which exhibited exceptional electrocatalytic performance for CO_2_ to CO, achieving an ultra-high current density of 1.23 A cm^−2^ at–2.4 V vs. RHE and remarkable durability (Fig. 8f). The mechanism study showed that the introduction of axial N-coordination can improve the degree of electron delocalization on the active site surface and lead to a distinct Fe 3d orbital splitting in contrast to the classical Ni–N_4_ structure. Additionally, a mesoporous nanosphere-supported catalyst with axial Co–N_5_ coordination and hierarchical pore structure was synthesized [173]. A series of experiments and theoretical calculations demonstrated that the initiation of localized d–p orbital hybridization by axial N-coordination can effectively enhance the oxidation state of Co. When combined with an optimized pore structure, the catalyst exhibited significantly improved performance in CO_2_RR.

#### Oxygen (O) Ligand Axially Coordinated SACs for CO_2_RR

**Fig. 9:**
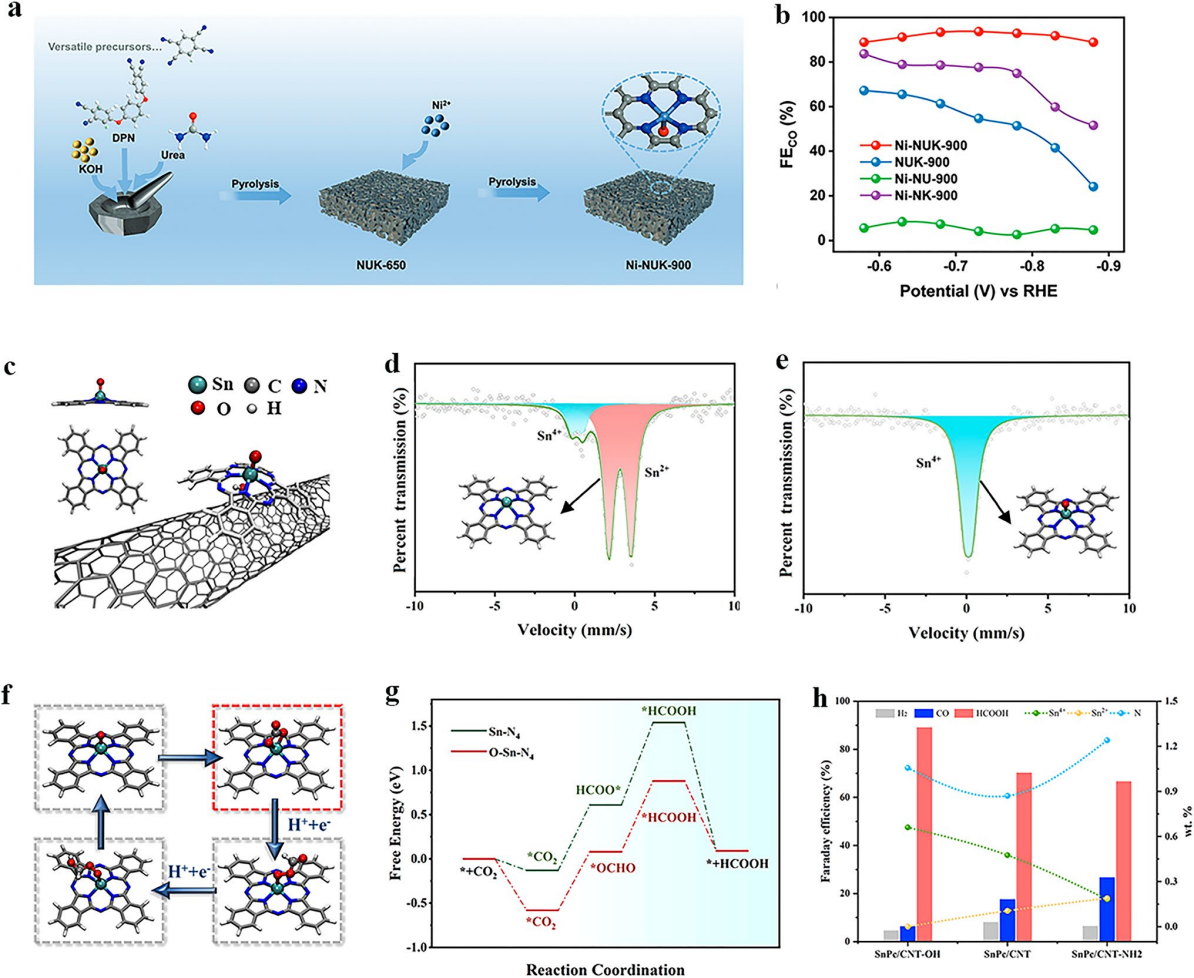
**a** Schematic diagram of the synthetic process of Ni-NUK-900. **b** FE_CO_ of Ni-NUK-900, NUK-900, Ni-NU-900, and Ni-NK-900 at different applied potentials. Reproduced with permission [162]. Copyright: 2023 Wiley-VCH GmbH. **c** Schematic illustration for the preparation of SnPc/CNT-OH. **d**, **e** Room-temperature Sn Mossbauer spectra for SnPc and SnPc/CNT-OH, respectively. **f** Proposed reaction pathway for CO_2_RR over the O–Sn–N_4_ site. g Calculated Gibbs free energy diagrams for CO_2_RR to HCOOH over O–Sn–N_4_ and Sn-N_4_ sites, respectively. **h** Correlation diagram of the CO_2_RR Faradaic efficiency and the relative contents of Sn(II), Sn(IV), and nitrogen species of SnPc/CNT, SnPc/CNT-OH, and SnPc/CNT-NH_2_: FE is indicated as column, while Sn(II), Sn(IV), and N contents are indicated as curves. Reproduced with permission [166]. Copyright: 2023 American Chemical Society

The electronic structure of the metal central site can likewise be tuned by introducing an axial coordination of O atoms, and thus improving the activity of SACs in CO_2_RR. With higher electronegativity of O than N, SACs axially coordinated with O ligands exhibit even greater performance enhancement compared to those coordinated with axial N ligands. The metal atoms of Ni–N_4_ surrounded by four pyridinic N atoms can introduce additional axial O coordination through various synthetic strategies. Several studies have demonstrated that due to the adjustment of the local geometry and electronic structure of the central atom by axial O ligands, the adsorption and activation of CO_2_ on the Ni site have been improved, resulting in changes in formation and adsorption energy barriers for various intermediates, ultimately leading to enhanced CO_2_RR performance [159–161, 174]. Employing KOH as the O source, pore-forming agent, and promoter, a SAC with O axial coordination, denoted as Ni-NUK-900, was successfully synthesized, as depicted in Fig. 9a [162]. The XRD and Raman spectra confirmed the atomically dispersed Ni, and the coordination configuration was identified as Ni–N_4_–O through EXAFS curve fitting. Ni-NUK-900 displayed excellent CO_2_RR performance with a high FE_CO_ of 94% and TOF_CO_ of 11,362 h^−1^ (Fig. 9b). DFT calculations demonstrated that the axially coordinated O atom acts as an electronic regulator at the Ni site, thus optimizing the formation of *COOH and the desorption of *CO. Moreover, extensive research has been made by researchers to explore the Fe–N_4_ structure [175], and both experimental and theoretical calculations confirmed the analogous contribution of axial O atoms in enhancing the catalytic CO_2_RR performance [163, 164]. Chen et al. [165] proposed a fast-pyrolyzing and controllable-activation strategy to synthesize the atomically dispersed Fe–N_4_ site with axial O coordination (Fe_1_N_4_–O_1_), which exhibited nearly 100% FE_CO_ across a broad potential range. DFT calculations revealed that the lower occupancy of the antibonding state of the adsorbed species and Fe_1_N_4_–O_1_ effectively regulated the binding interaction of CO_2_RR intermediates. The calculation results further confirmed that the axial O atom with high electronegativity contributed to superior performance of the catalyst in promoting CO_2_RR and inhibiting HER.

In a study by Zhao Li et al. [166], the incorporation of O into an Sn–N–C catalyst was investigated. They developed a series of Sn-SACs with well-controlled coordination and electronic structure to examine the activity of reducing CO_2_ to HCOOH. The room-temperature Sn Mossbauer spectra revealed that the introduction of the O ligand results in a complete conversion of Sn(II) to Sn(IV), with the percentage reaching 100% (Fig. 9d, e). According to electrochemical performance test data, the ratio of Sn(IV) and Sn(II) positively correlates with the ability of catalyst for HCOOH and CO formation, suggesting Sn(IV) species are most likely the main catalytic sites for CO_2_RR to HCOOH (Fig. 9h). Theoretical research confirmed that the O–Sn–N_4_ species can adjust the adsorption configuration of *CO_2_ by increasing the asymmetric distribution of Sn(IV) orbital electrons, which reduced the energy barrier for *OCHO species formation and hydrogenation, thereby promoting the conversion of CO_2_ to HCOOH (Fig. 9g).

Although many studies have validated the practicality of using axial O coordination strategies to enhance the CO_2_RR performance of catalysts, conventional methods pose challenges in achieving precise control over the type and content of O atoms. Therefore, it is imperative to explore the approach of incorporating O axial ligands more accurately. Moreover, the activity of the SACs hinges on the intrinsic characteristics of their constituent metal atoms [176]. Thus, selecting the appropriate metal atoms is equally important in obtaining high-performance M–N_4_O_1_–C SACs for CO_2_RR.

#### Sulfur (S) Ligand Axially Coordinated SACs for CO_2_RR

**Fig. 10:**
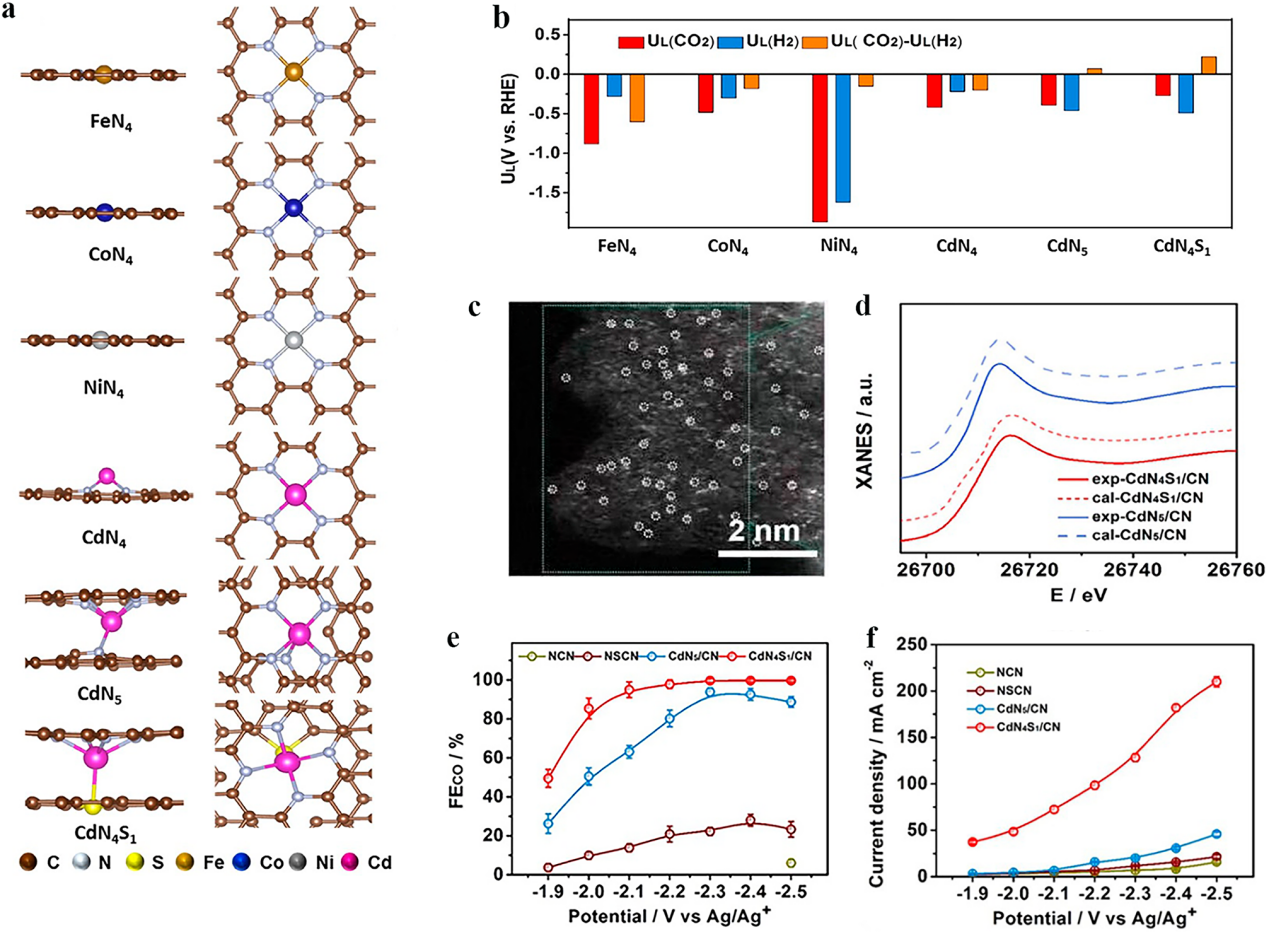
**a** The side and top views of FeN_4_, CoN_4_, NiN_4_, CdN_4_, CdN_5_, and CdN_4_S_1_ models. **b** Difference in limiting potentials for CO_2_ reduction and H_2_ evolution over different models. **c** HAADF-STEM image of CdN_4_S_1_/CN. **d** Comparison between the experimental and theoretically simulated XANES spectra of CdN_4_S_1_/CN and CdN_5_/CN. **e** FEs of CO for NCN, NSCN, CdN_5_/CN, and CdN_4_S_1_/CN at different applied potentials. **f** The total current densities for NCN, NSCN, CdN_5_/CN, and CdN_4_S_1_/CN at different applied potentials. Reproduced with permission [167]. Copyright: 2021 Wiley-VCH GmbH

S atoms possess high spin density and charge delocalization, which can effectively lower the free energy barriers for intermediates sorption during CO_2_RR [177]. Therefore, introducing S atoms as axial ligands into SACs could be a promising approach to enhance their catalytic performance. To investigate the synergistic effects of metal atoms and axial coordination structures on CO_2_RR electrocatalysis, Wu et al. constructed various SACs models of diverse metals for DFT simulation calculations [167]. Notably, for Cd-SAC with relatively large atomic size of Cd, they were observed to be situated in the upper region of the graphene layer within the constructed SACs model (as depicted in Fig. 10a). Based on this, the author proposed a reasonable assumption that Cd atoms could potentially coordinate axially with N or S atoms on adjacent graphite layers. In general, the value of (*U*_*L*_(CO_2_) – *U*_*L*_(H_2_)) can serve as an indicator of catalyst selectivity. Specifically, a greater positive difference results in higher selectivity for CO_2_RR on the corresponding catalyst and weaker competitiveness with HER. As depicted in Fig. 10b, CdN_4_S_1_ exhibits the most positive (*U*_*L*_(CO_2_) – *U*_*L*_(H_2_)) value among all constructed models, indicating its potential for optimal CO_2_RR selectivity. Guided by these theoretical calculations, Cd was selected as the active metal and S as the axial coordination atom, the CdN_4_S_1_/CN catalyst was successfully prepared by calcining amine, hydroxylamine hydrochloride, cadmium chloride, and L-cysteine together in an N_2_ atmosphere. The HAADF-STEM images revealed a homogeneous distribution of individual Cd atoms (Fig. 10c). Furthermore, comparison between the experimental and theoretically simulated XANES spectra confirm that the structure of the as-prepared catalyst is in agreement with the theoretical model (Fig. 10d). The FEco and current density of CdN4S1/CN were found to be higher than those of CdN5/CN at the overall potentials, which aligns well with the theoretical calculation results (Fig. 10e, f). The FEco and current density of CdN_4_S_1_/CN were found to be higher than those of CdN_5_/CN at the overall potentials, which aligns well with theoretical calculation results (Fig. 10e, f).

Previous study has demonstrated that introducing P atoms into N-doped carbon materials to form P stabilized at various coordination shells around the central metal atom can effectively decrease the electronic density of metal sites. This significantly reduces the formation energy barrier of *COOH, resulting in high CO_2_RR performance at low overpotential [178]. Inspired by this, Hu and co-workers [168] employed a “synergistically near- and long-range regulation” strategy to fabricate a ZnN_4_S_1_/P-HC catalyst. This catalyst features an axial S ligand and is surrounded by P atoms in the carbon matrix, which exhibited excellent CO_2_RR performance across a wide potential range, achieving over 90% FE_CO_ with nearly 100% FE_CO_ at −0.6 V vs. RHE. DFT calculations further confirmed that the electronic structures were optimized and the interaction between Zn–N_4_ active sites and *COOH was greatly enhanced by synergistic regulation from axially coordinated S and long-range P atoms, which greatly enhanced the electrocatalytic CO_2_RR performance. Additionally, a fascinating instance has been observed in which the S atom in diphenyl sulfide was axially coordinated with Co-SACs that were anchored on graphene [179]. The benzene ring of diphenyl sulfide exhibited a strong face-to-face stacking with graphene, and the axial coordination atoms acted as relay molecules to promote interfacial electronic exchange, thus further improving the CO_2_RR activity of the catalyst.

#### Halogen (Cl, Br, I) Ligand Axially Coordinated SACs for CO_2_RR

In the article reported by Li et al. [169], they demonstrated an example of incorporating axial Cl ligand into an FeN_4_Cl/NC catalysts through a two-step method involving pyrolysis and low-temperature hydrochloric acid incubation. The Fe single atoms exhibit in-plane coordination with four N atoms and one axial Cl ligand. It was found that the FE_CO_ of FeN_4_Cl/NC is much higher than the counterpart FeN_4_/NC over a wide potential range. Additionally, a high current density of 10.8 mA cm^−2^ was achieved at a low overpotential of 490 mV. DFT calculations revealed that electrons transfer from the axial Cl atom to the central Fe atom, causing a negative shift of the *d-band* center of FeN_4_Cl. The reduction in the *d-band* weakens the bonding interaction between adsorbed species, which favors *CO desorption and suppresses *H adsorption, leading to a higher FE_CO_. Zhang et al. [170] successfully synthesized an Mn-based heterogeneous catalyst through Cl and N dual-coordination tactics. The axial Cl coordination-induced distortion in the single atom Mn center facilitated the adsorption of CO_2_ and *COOH, leading to a stable low-energy transition state that promotes final CO desorption. This modification resulted in a maximum FE_CO_ of 97% at 0.49 V vs RHE and an increased partial current density.

**Fig. 11:**
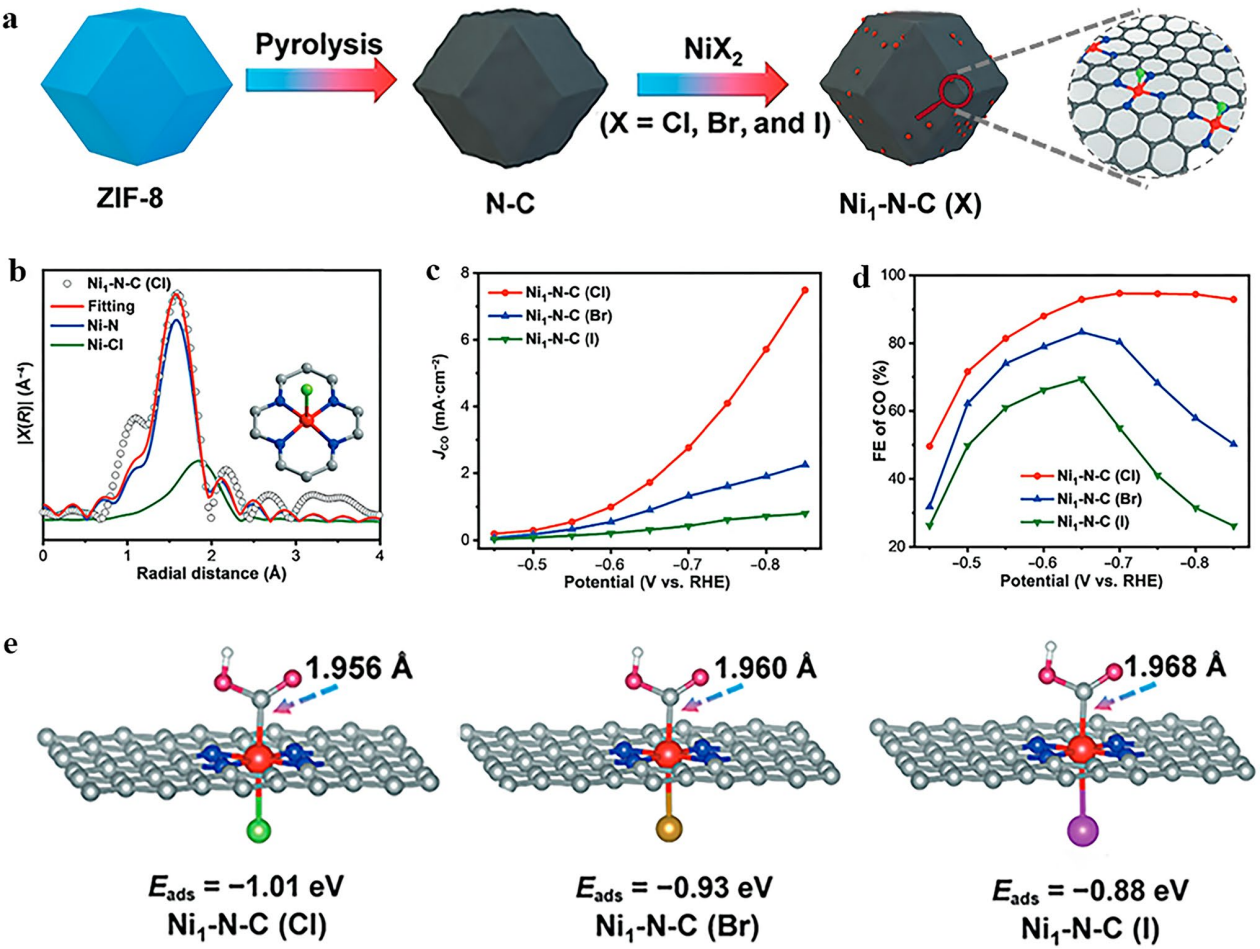
**a** Illustration for the construction of Ni_1_–N–C(X) (X = Cl, Br, and I) single-atom catalysts via a PMHM strategy. **b** EXAFS fitting of Ni_1_–N–C(Cl) (inset: optimized coordination configuration of Ni atom). **c** CO partial current densities, and **d** CO FEs of Ni1–N–C(X) (X = Cl, Br, and I). **e** DFT-optimized configurations of Ni_1_–N–C(X) (X = Cl, Br, and I) with *COOH adsorption and corresponding adsorption energy. Reproduced with permission [171]. Copyright: 2022, Tsinghua University Press

Axial halogen atoms with distinct electronegativity can perturb the charge distribution in the original plane of SACs and modulate the electronic state of the central atoms. A strategy of post metal halide modification (PMHM) was developed to precisely adjust the axial coordination environment of SACs at the atomic level. Based on this, Peng et al. [171] synthesized a series of Ni–N–C (X) (X = Cl, Br, I) materials with different halogen axial coordination (Fig. 11a, b). By combining experimental data with theoretical calculation, the crucial role of halogen axial coordination in CO_2_RR has been systematically elucidated. Ni–N–C (Cl) possessed high CO partial current densities and a CO selectivity of up to 94.7% in CO_2_RR, surpassing those Ni–N–C catalysts axially coordinated with Br and I (Fig. 11c, d). Through theoretical calculations, it was discovered that the axial halogen atom can facilitate the formation of intermediate *COOH, thereby accelerating CO_2_RR for CO generation. The variation in electron delocalization degree of Ni atoms caused by the distinct electronegativity of axial halogen ligands is believed to impact the energy barrier of the reaction. For the three different axial halogen ligands studied in this work, it is evident that the electronegativity of axial halogen ligands follows the order Cl > Br > I. Therefore, it can be reasonably inferred that Ni transfers more electrons to Cl than Br and I, and Cl axial ligand coordinated Ni–N–C catalyst exhibited the lowest energy barrier for CO_2_RR. To confirm this perspective, the free energy change of CO_2_ reduction to CO was computed. In the process of CO_2_ reduction to CO, the formation of *COOH is commonly regarded as a rate-limiting step. The calculation results showed that the adsorption energy of *COOH follows the sequence Ni–N–C (Cl) < Ni–N–C (Br) < Ni–N–C (I) for SACs with different axial halogen coordination (Fig. 11e), which is consistent with the electronegativity order of relevant halogen atoms. Meanwhile, there were fewer localized electrons between Ni–N–C (Cl) and *CO. The above findings suggest that the interaction between Ni–N–C (Cl) and *COOH is strong, while the interaction with *CO is relatively weak. The fine-tuned intermediate adsorption behavior results in the superior CO_2_RR performance of Ni–N–C (Cl).

#### Other Ligands Axially Coordinated SACs for CO_2_RR

With the modification of axial ligands on SACs, the rate-determination step (RDS) of CO_2_RR for CO generation can shift from a non-electrochemical step (CO desorption) to an electrochemical control step. This opens up possibilities for optimizing catalyst performance by regulating the applied voltage, pH, and other reaction conditions. The electronic energy levels of central metal atoms can be effectively optimized by the appropriate acquisition or loss of electrons by axial ligands. Due to the differences in the electronegativity of the ligands introduced, metal atom sites experience varying degrees of electron loss. Experiments have shown that M–N–C SACs with axial coordination exhibit remarkable activity in CO_2_RR, yet the current literature predominantly concentrates on ligands containing N, O, S, and halogen. Since diverse organic ligands have been employed for the stabilization of metal nanoclusters and nanoparticles. Therefore, in principle, it can also be applied to metal SACs through covalent grafting. Ma et al. [180] investigated a range of axial ligands including both common heteroatom-based ligands and organic ligands for the functionalization of Fe–N_4_/Gra, Co–N_4_/Gra, and Ni–N_4_/Gra SACs. Theoretical calculation results confirmed the significant optimization of axial organic ligands for Fe–N_4_/Gra and Co–N_4_/Gra SACs. Conversely, the selectivity of Ni–N_4_/Gra SAC is weakened by most axial ligands. Liu et al. [181] have systematically studied more than 20 p-block elements as axial ligands to tailor the Mo–N_4_ structure for CO_2_RR. Theoretical calculations revealed the possibility of using p-block elements as axial ligands to improve the performance of the catalyst for two-electron CO_2_RR. In contrast to conventional findings, the conclusion of this study highlighted the exceptional performance of Ge–MoN_4_ structure in two-electron CO_2_RR, providing theoretical evidence for the feasibility of utilizing metal atoms for axial coordination. This offers guidance for the rational design of SACs towards electrocatalytic CO_2_RR.

### Hydrogen Evolution Reaction

Electrocatalytic water splitting for hydrogen evolution is considered the best way for green hydrogen production due to its advantages including high energy conversion efficiency, cleanliness, and zero-[182, 183]. During the process, the cathode undergoes the hydrogen evolution reaction (HER), in which H^+^ or H_2_O obtains two electrons to generate H_2_ gas [184]. There are two predominant pathways for HER, namely the Volmer–Tafel mechanism and the Volmer–Heyrovsky mechanism, which involve subsequent processes.

Volmer step:

$${\text{H}}^{ + } + {\text{e}}^{ - } \to \;^{*} {\text{H }}({\text{in acidic electrolyte}})$$

$${\text{H}}_{{2}} {\text{O}} + {\text{e}}^{ - } \to {\text{OH}}^{ - } + \;^{*} {\text{H }}({\text{in alkaline electrolyte}})$$

Tafel step:

$$\;^{*} {\text{H}} + \;^{*} {\text{H}} \to {\text{H}}_{{2}} ({\text{in acidic and alkaline electrolyte}})$$

Heyrovsky step:

$$\;^{*} {\text{H}} + {\text{H}}^{ + } + {\text{e}}^{ - } \to {\text{H}}_{{2}} ({\text{in acidic electrolyte}})$$

$${\text{H}}_{{2}} {\text{O}} + {\text{e}}^{ - } \to {\text{H}}_{{2}} + {\text{OH}}^{ - } ({\text{in alkaline electrolyte}})$$

However, this process necessitates crossing high-energy barriers that require catalysts to effectively reduce activation and reaction energies. Therefore, it is crucial to develop HER electrocatalysts with robust performance. SACs have emerged as a new frontier in HER catalysis due to their exceptional atomic utilization, selectivity, and catalytic performance [185]. Both theoretical comprehension and experimental validation have confirmed that SACs exhibit superior performance compared to traditional heterogeneous catalysts in HER catalysis [186]. The synthesis of SACs has attracted widespread attention in HER electrocatalysis. Previously, researchers were mostly committed to regulating the interaction between supports and metal atoms, screening carriers with unique properties, and pursuing the enhancement of single-atom active site loading [187]. In recent years, axial coordination optimization strategy has emerged as a prominent field in the structural design of SACs (Table 3). This section presents an overview of recent advances in the application of SACs modified with axial coordination for HER.

**Table 3:** Summarized electrochemical HER and OER activity of typical axially coordinated SACs

Axial-coordinated SACs	Coordination structures	Axial atom	Reaction	Electrolyte	*η*_10_ (mV)	Tafel slope (mV dec^−1^)	Refs
Pt–GDY1	C_1_–PtCl_4_	C	HER	0.5 M H_2_SO_4_	113	52.0	[188]
NiN_4_–Cl SAs/N–C	NiN_4_–Cl	Cl	HER	1.0 M KOH 0.5 M H_2_SO_4_	243 274	89.2 96.4	[189]
CoN_4_–O/MX	CoN_4_–O	O	OER	1.0 M KOH	350	–	[199]
V@NMCNFs	V–O_2_N_3_	O	OER	0.5 M H_2_SO_4_	196	25	[200]
P–CoPc@CNT	Co_1_N_4_–PO_4_	P	OER	1.0 M KOH	300	41.7	[201]

**Fig. 12:**
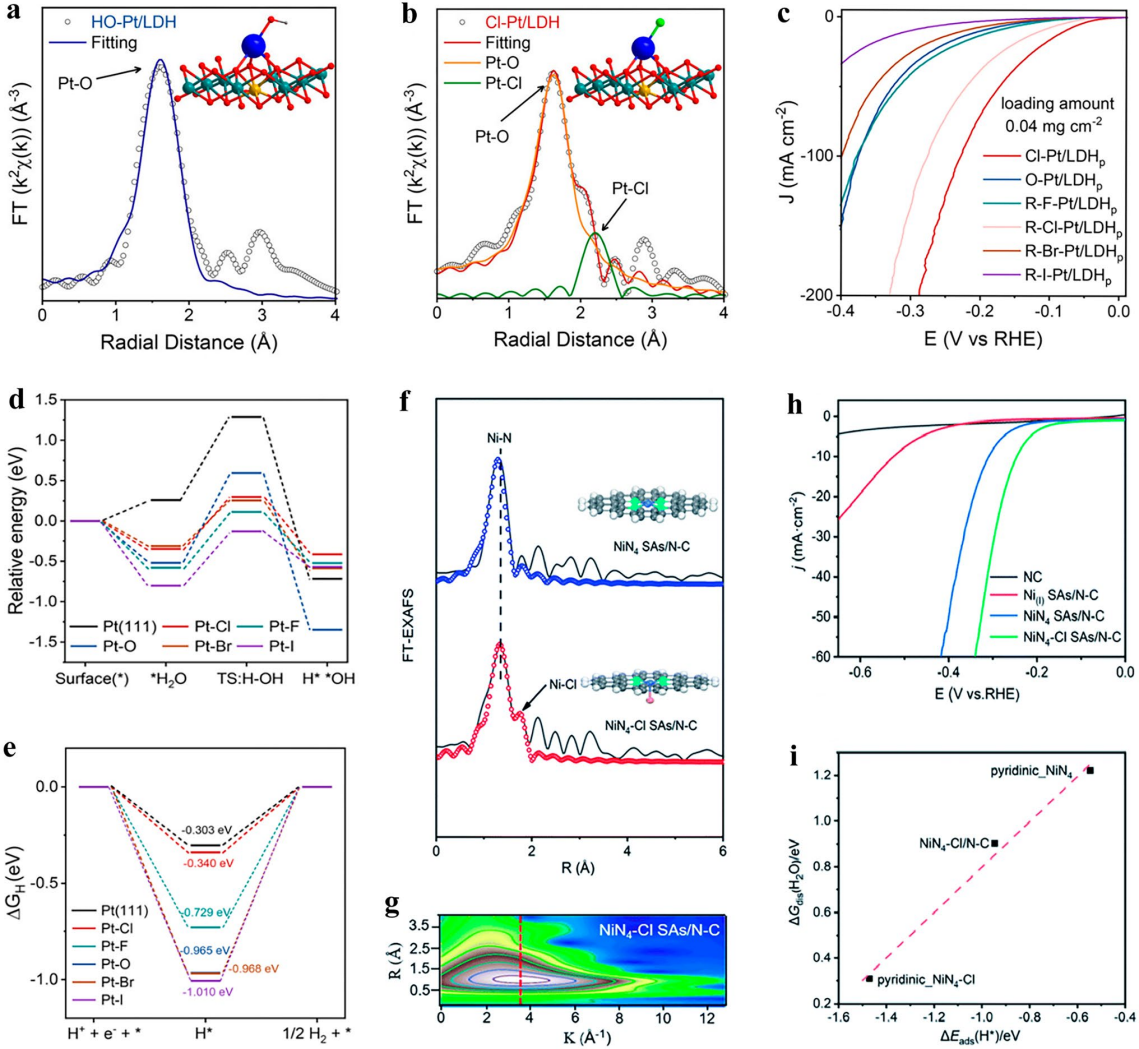
EXAFS fitting curve (inset is the magnified local structure) of **a** Cl–Pt/LDH and **b** HO–Pt/LDH**,** the blue, olive, yellow, red, green, and gray spheres refer to Pt, Ni, Fe, O, Cl, and H atoms, respectively. **c** HER polarization curves of the Pt-SACs with different axial ligands. Calculated energy barriers of **d** water dissociation kinetics and **e** adsorption free energies of *H on the surfaces of the Pt-SACs, and the Pt (111) slab as a reference. Reproduced with permission [77]. Copyright: 2022, Springer Nature. **f** R space fitting diagram and theoretical spectra (the greyish-black, dark-green, blue, and orange balls refer to C, N, Ni, and Cl atoms, respectively). **g** Wavelet transform diagram of NiN4–Cl SAs/N–C. **h** HER polarization curves of NC, Ni(I) SAs/N–C, NiN4 SAs/N–C, and NiN4–Cl SAs/ N–C in 1 M KOH. **i** Illustration of the linear correlation between Δ*G*_dis_(*H_2_O) and Δ*E*_ads_(*H). Reproduced with permission [189]. Copyright: 2022, Royal Society of Chemistry. (Color figure online)

Among the numerous developed electrocatalysts, the excellent HER performance of Pt-based catalysts has been verified. Whereas, in consideration of the scarcity and high cost of noble-metal Pt, developing Pt-SACs and further optimizing their performance is necessary for electrochemical HER in large-scale applications. Therefore, an axial coordination strategy has been attempted by Zhang et al. [77], they synthesized Pt-SACs by anchoring Pt single atom onto NiFe-layered-double-hydroxides (NiFe-LDH). Meanwhile, a facile irradiation-impregnation procedure was employed to achieve a range of heteroatoms axially coordinated Pt-SACs, the final materials are denoted as X-Pt/LDH (X = F, Cl, Br, I, and OH) (Fig. 12a, b). This work explored the impact of various axial ligands with distinct electron affinities on Pt centers in Pt-SACs. The HER activity of X-Pt/LDH was found to follow the order of Cl–Pt/LDH > F–Pt/LDH > HO–Pt/LDH > Br–Pt/LDH > I–Pt/LDH under identical experimental conditions, suggesting the HER activity of Pt-SACs is significantly influenced by axial ligands coordination (Fig. 12c). Among these modified Pt-SACs, the Cl ligand axially coordinated Pt site exhibited optimal adsorption affinity for both *OH and *H due to the higher first electron affinity of Cl axial ligand, thereby promoting the kinetic limiting Volmer step for H_2_O dissociation in alkaline HER (Fig. 12d, e). Moreover, there have been investigations on the axial coordination of C ligand to the metal center of SACs for HER. Yin et al. [188] investigated two Pt-SACs, the Pt-GDY1 owns five non-planar-coordinated C_1_–Pt–Cl_4_ configuration, and the Pt-GDY2 with four-coordinated C_2_–Pt–Cl_2_ configuration, which have the same ligand atom species but different configurations. Electrochemical testing results showed that Pt-GDY2 exhibited higher electrocatalytic HER activity compared to Pt-GDY1, with a more than threefold increase in mass activity. The performance difference indicates the importance of reasonable design of coordination configuration for improving the HER activity of SACs after determining the ligand atoms.

Apart from Pt-SACs, researchers have also explored axial optimization strategies for Ni-SACs. Li et al. [189] successfully constructed NiN_4_–Cl active sites via a doping-adsorption-pyrolysis strategy. R-space EXAFS fitting and wavelet transform jointly demonstrate the presence of an axial Ni–Cl bond at the active site of NiN_4_–Cl_1_, and the change in coordination environment of the central Ni atom (Fig. 12f, g). The synthesized NiN_4_–Cl SAs/N–C catalyst exhibited higher HER activity, lower overpotential, and enhanced long-term stability compared to NiN_4_ without axial coordination_._ To achieve the current density of 10 mA cm^−2^ in alkaline electrolyte, a low overpotential of 243 mV is required for NiN_4_–Cl SAs/N–C (Fig. 12h). DFT calculations were applied to explain the role of axial Cl ligands in this catalyst. It indicated that the axial Cl coordination in NiN_4_–Cl SAs/NC induces increased electron localization, which facilitates the adsorption and activation of *H intermediates, thereby accelerating the HER process (Fig. 12i).

### Oxygen Evolution Reaction

The electrochemical oxygen evolution reaction (OER) is crucial in various energy conversion and storage devices, such as water electrolysis, metal-air batteries, and fuel cells [30]. The OER involves the conversion of water into oxygen gas and protons, which is a thermodynamically uphill process and requires activation energy to proceed. Its efficiency and performance are essential in determining the overall efficiency and performance of these devices. Therefore, research efforts aimed at improving the efficiency of the OER are crucial in advancing the development of sustainable energy technologies. The reaction mechanisms for OER in both acidic and alkaline solutions are as follows [190]:

In acidic medium:

$${\text{H}}_{{2}} {\text{O}} + * \to {\text{OH}}_{{{\text{ads}}}} + {\text{H}}^{ + } + {\text{e}}^{ - }$$

$${\text{OH}}_{{{\text{ads}}}} \to {\text{O}}_{{{\text{ads}}}} + {\text{H}}^{ + } + {\text{e}}^{ - }$$

$${\text{O}}_{{{\text{ads}}}} + {\text{H}}_{{2}} {\text{O}} \to {\text{OOH}}_{{{\text{ads}}}} + {\text{H}}^{ + } + {\text{e}}^{ - }$$

$${\text{OOH}}_{{{\text{ads}}}} \to {\text{O}}_{{{\text{2ads}}}} + {\text{H}}^{ + } + {\text{e}}^{ - }$$

$${\text{O}}_{{{\text{2ads}}}} \to {\text{O}}_{{2}} + *$$

$${\text{2H}}_{{2}} {\text{O}} \to {\text{O}}_{{2}} + {\text{4H}}^{ + } + {\text{4e}}^{ - } ({\text{overall reaction}})$$

In alkaline medium:

$${\text{OH}}^{ - } + * \to {\text{OH}}_{{{\text{ads}}}} + {\text{e}}^{ - }$$

$${\text{OH}}_{{{\text{ads}}}} + {\text{OH}}^{ - } \to {\text{O}}_{{{\text{ads}}}} + {\text{H}}_{{2}} {\text{O}} + {\text{e}}^{ - }$$

$${\text{O}}_{{{\text{ads}}}} + {\text{OH}}^{ - } \to {\text{OOH}}_{{{\text{ads}}}} + {\text{e}}^{ - }$$

$${\text{OOH}}_{{{\text{ads}}}} + {\text{OH}}^{ - } \to {\text{O}}_{{{\text{2ads}}}} + {\text{H}}_{{2}} {\text{O}} + {\text{e}}^{ - }$$

$${\text{O}}_{{{\text{2ads}}}} \to {\text{O}}_{{2}} + *$$

$${\text{4OH}}^{ - } \to {\text{O}}_{{2}} + {\text{2H}}_{{2}} {\text{O}} + {\text{4e}}^{ - } ({\text{overall reaction}})$$

**Fig. 13:**
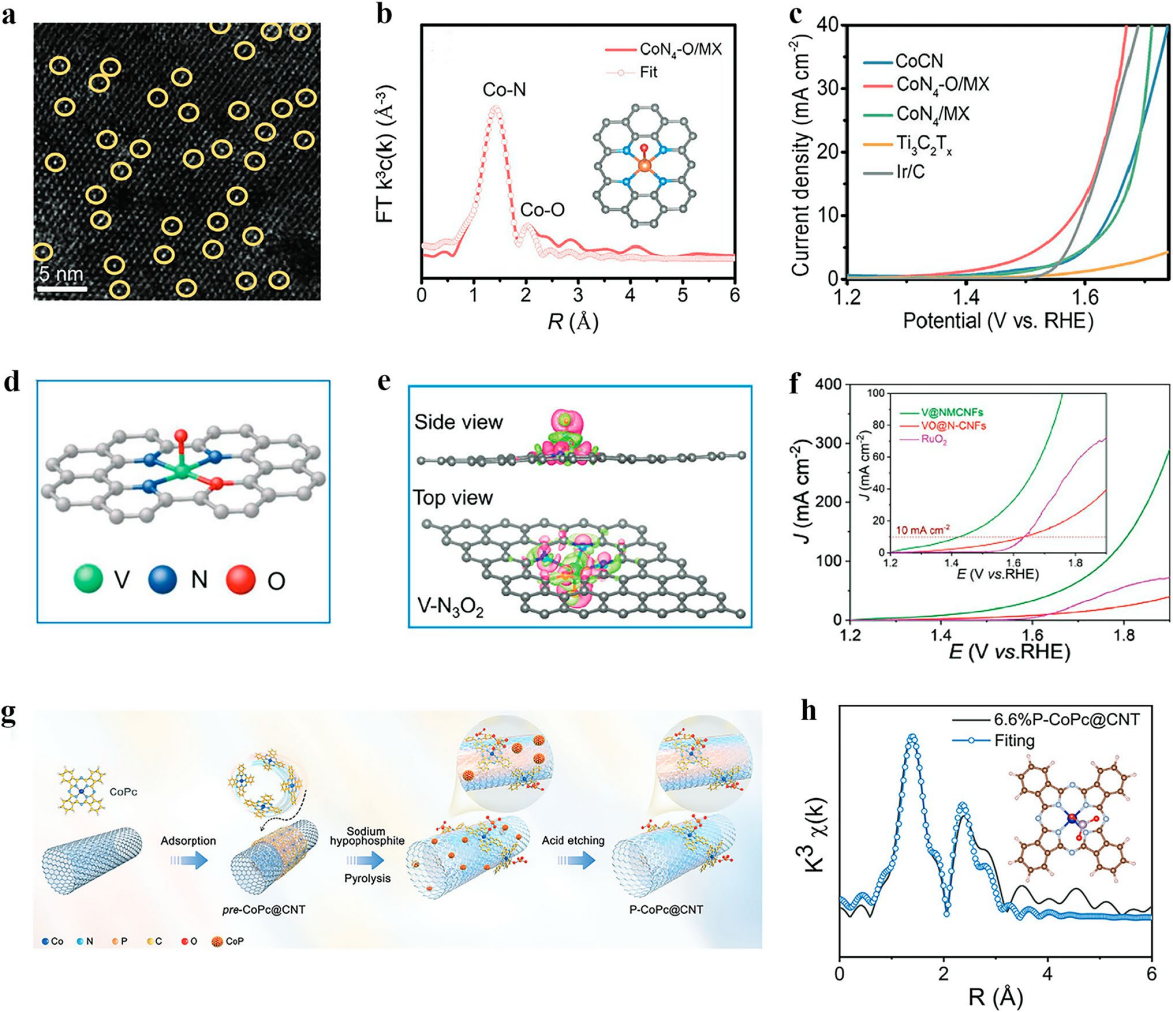
**a** HADDF-STEM image of CoN_4_–O/MX. **b** The EXAFS fitting curve in R-space for CoN_4_–O/MX. **c** LSV curve of CoN_4_–O/MX for OER. Reproduced with permission [199]. Copyright: 2022, Wiley-VCH GmbH. **d** Schematic atomic structure model of the proposed V–N_3_O_2_ configuration. **e** Differential charge density distributions for V–N_3_O_2_ (pink and light green colors represent charge accumulation and depletion, respectively). **f** LSV polarization curves of V@NMCNFs with other reported electrocatalysts. Reproduced with permission [200]. Copyright: 2022, Wiley-VCH GmbH. **g** Schematic illustration for the synthesis of 6.6%P-CoPc@CNT. **h** The corresponding EXAFS fitting curve of 6.6%P-CoPc@CNT in R space. Inset: Schematic model of 6.6%P-CoPc@CNT (C: brown, N: gray, Co: blue, P: mauve, O: red). Reproduced with permission [201]. Copyright: 2022, Wiley-VCH GmbH. (Color figure online)

Generally, OER is plagued by high overpotential and sluggish kinetics, so it is necessary to develop highly active electrocatalysts [191, 192]. Various catalysts have been extensively investigated for their potential in electrocatalytic OER [193–196], wherein SACs are widely employed due to their unique structural characteristics and high efficacy. However, smaller particles tend to aggregate into clusters or particles due to their high surface energy [197]. Therefore, various strategies have been studied to make SACs as dispersed and stable as possible. Among them, axial coordination as a new strategy is gradually coming into view. Deng et al. [198] investigated the 2D transition metal-based tetracyanoquinodimethane (TM-TCNQ, TM = Cr, Cu, Ru, Ag, Pt, Ir) with single atom site structures for OER via DFT calculations. The Fe-TCNQ-Cl and Fe-TCNQ-CO catalysts, which respectively have axial coordination of Cl and CO in Fe-TCNQ material, exhibited higher OER activity and lower overpotential compared to pristine Fe-TCNQ, affirming the potential of axial coordination for improving OER performance. Currently, very few axial coordination designs have been reported as effective strategies for SACs used in OER (Table 3), and there is still significant research potential and promising prospects for further exploration of this strategy. For instance, Zhang et al. [199] incorporated axial O ligand into Co–N_4_ single atomic site to form a Co–N_4_O_1_ configuration on MXene nanosheets (CoN_4_–O/MX). The AC HADDF-STEM image of CoN_4_–O/MX showed abundant bright isolated spots on the MXene substrate (highlighted with yellow circles), indicating the presence of atomically dispersed Co atoms (Fig. 13a). Additionally, the researchers conducted a least-squares fitting analysis of EXAFS on the catalyst, revealing the coexistence of Co–N and Co–O coordination bonds in CoN_4_–O/MX, with respective coordination numbers of 4.0 and 1.0. This finding confirmed the axial regulation of the CoN_4_–C active site by the epoxy group on surface of MXene (Fig. 13b). The CoN_4_–O/MX catalyst exhibited outstanding OER performance, achieving a potential of 1.55 V at 10 mA cm^−2^ (Fig. 13c). This surpasses the performance of other catalysts and highlights the positive impact of axial O regulation on enhancing OER activity. Similarly, Li et al. [200] successfully constructed atomically dispersed V sites with O axial coordination on N-doped multi-channel carbon nanofibers support (V@NMCNFs). Based on both experimental and theoretical calculations, it was proposed that the configuration of central V atom in V@NMCNFs is a five-coordinated V-N_3_O_2_ moiety with axial O atom coordination (Fig. 13d). The analysis of charge density difference revealed significant charge accumulation on the V atom of the V-N_3_O_2_ moiety, leading to charge depletion on adjacent carbon substrates, thereby improving the charge transfer ability and conductivity of the V-N_3_O_2_ moiety (Fig. 13e). The V@NMCNFs catalyst demonstrated superior OER activity compared to the reference samples (VO@N-CNFs and commercial RuO_2_ benchmark), exhibiting the highest OER activity with the lowest overpotential and largest current response (Fig. 13f). This makes it one of the most competitive OER electrocatalysts among non-noble metals reported to date.

In addition to O ligands, other ligands have also been excavated to be axially coordinated with SACs to improve their OER electrocatalytic activity. Liu et al. [201] achieved axial PO_4_ coordination at the Co_1_N_4_ site of cobalt phthalocyanine adsorbed on carbon nanotubes (P-CoPc@CNT) to enhance OER activity. They synthesized the P-CoPc@CNT catalyst by mixing CoPc@CNT with excess sodium hypophosphite (NaH_2_PO_2_) followed by pyrolysis and acid etching (Fig. 13g). The EXAFS spectrum of 6.6% P-CoPc@CNT was analyzed in R space, and the bond lengths corresponding to the two characteristic peaks were 1.92 and 2.49 Å (phase shift correction), respectively (Fig. 13h). The bond lengths are consistent with the planar Co–N and axial Co–O bond present in the CoPc-PO_4_ model, providing further evidence for the axial coordination structure of 6.6% P-CoPc@CNT. The 6.6% P-CoPc@CNT catalyst with tailored axial PO_4_ group exhibited excellent OER performance, with a low overvoltage of 300 mV and a Tafel slope of 41.7 mV dec^−1^, which is significantly superior to the CoPc@CNT without axial PO_4_ coordination. Apart from experimental approaches, DFT calculations had also been utilized to study the impact of axial PO_4_ ligands on OER performance. The results revealed that after coordinating axial PO_4_ ligands, the adsorption strength of reaction intermediates on central Co sites can be optimized, thereby improving the OER activity of the catalyst.

### Nitrogen/Nitrate Reduction Reaction

Axial coordination design of SACs has also been applied to other electrochemical reactions, such as NO_3_^−^ reduction reactions (NO_3_RR) and electrochemical nitrogen reduction reaction (NRR) [202]. The electrocatalytic activity of Fe–N_4_–C catalysts for NO_3_RR was investigated through DFT calculations, focusing on the impact of various ligands axially coordinated at the central Fe atoms [203]. It has been found that the axial coordination of ligand X (X = O, OH, F, Cl, Br, I) to the Fe center of Fe–N_4_–C can significantly improve its catalytic activity in electrocatalytic reduction of NO_3_^−^ to NH_3_. Simultaneously, further investigation of the mechanism underlying its remarkable NO_3_RR activity confirmed that the significant catalytic activity is attributed to the orbital hybridization of Fe^3dxz^/Fe^3dyz^ and NO^π*^, and a moderate *NO adsorption free energy (ΔG_*NO_). In addition, when preparing a series of single-atom Fe/NC electrocatalysts through pyrolysis at different temperatures, Liu et al. [204] found that the active site of Fe_1_/NC-800 catalyst is OH axially coordinated Fe–N_4_ (a square-based cone with OH at the top). The axial coordination of OH disrupts the electronic balance of the FeN_4_ active site, thereby improving its NO_3_RR electrocatalytic performance to some extent. However, its NO_3_RR electrocatalytic activity does not exceed that of Fe_1_/NC-900 with an Fe–N_3_ triangular cone configuration, which showed excellent NO_3_RR performance with a FE of 86.7% and a yield rate of 18.8 mg_NH3_ h^−1^ mg_cat_^−1^. Although the attempt has been made on axial coordination design to enhance the electrocatalytic performance of SACs for NO_3_RR, it is evident that challenges remain in this field, and further exploration and efforts are required by researchers. Wu et al. [205] designed Co-SAs/N–C with a sixfold coordination structure by a tandem non-thermal plasma-electrocatalysis strategy, which achieved efficient N_2_ fixation to NH_3_ in NRR. The best-fit analysis of EXAFS data in R-space and k-space suggested four planar Co–N and two axial Co–O bonds in the first coordination shell of Co-SAs/N–C. DFT calculation results confirmed the superior impact of the axial coordination structure of the catalyst on *NH_3_ desorption and *H adsorption, providing compelling evidence for its exceptional catalytic performance. Although the axial coordination design of SACs in N-cycling electrocatalysis is largely unexplored, these endeavors hold significant reference value for enlightening the synthesis of high-value compounds through the utilization of other small molecules (e.g., NO, CO, CO_2_, CH_4_…).

## Summary and Perspectives

**Fig. 14:**
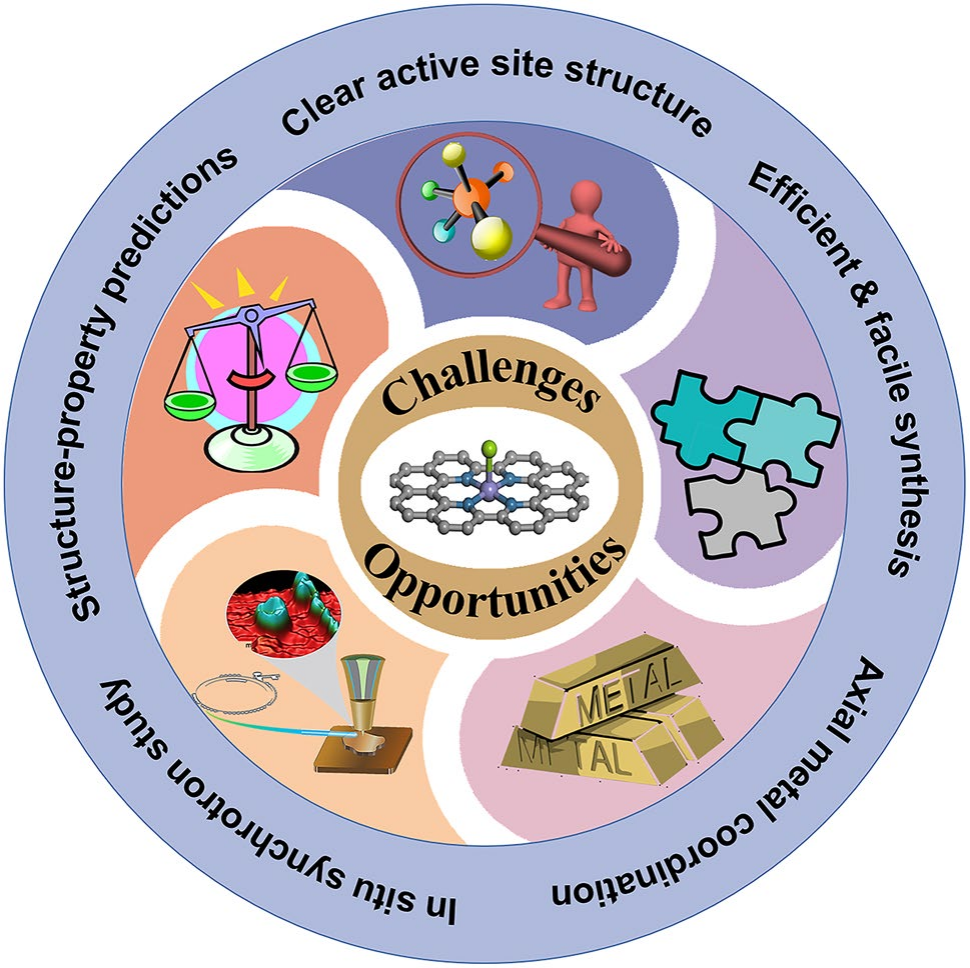
Illustration of the challenges and opportunities in the future development of axially coordinated singe-atom catalysts

We herein present a comprehensive review of the latest advances in the development of axial coordination design of SACs, covering their synthetic strategies to energy electrocatalysis applications. In this review, the efficient axial coordination synthetic strategies of SACs have been categorized and summarized, and their electrocatalytic performance as well as reaction mechanisms toward different electrochemical reactions have been overviewed. The high feasibility and promising potential of the axial coordination strategy of SACs to improve their activity and selectivity in electrocatalysis have been successfully demonstrated. It clearly elucidates the crucial roles played by axial ligands in modulating both the geometric and electronic structures of the metal single sites, endowing breakthroughs of knowledge in electrocatalytic SACs regarding the activity and reaction mechanism. However, considering the diverse couplings between axial ligands and metal single sites in SACs, there is great space for the exploration of axially coordinated SACs library tailored to various catalytic reactions with enhanced activity, selectivity and optimized reaction pathways. Consequently, the understanding of SACs will undoubtedly be further discovered and deepened. Despite significant advances and good prospects, axial coordination design for regulating the electrocatalytic activities and stabilities of SACs is still in its infancy. Therefore, challenges and opportunities exist in this rapidly developing field (Fig. 14).

(1) Although several synthetic methods have been reported for preparing axial ligand modified SACs, it is still a big challenge to achieve precise control of the exotic ligand to be axially coordinated to the single atom sites. Consequently, facile, efficient and generalized routes are expected to be developed to realize elaborate axial coordination design on SACs. Considering the complicated and uncontrolled synthetic processes for SACs, the post-modification on the axial position of a pre-synthesized planar SAC by chemical bonding or electrodeposition could be a promising route for the rational construction of the axially coordinated SACs. Moreover, the exploration of a comprehensive understanding of the principles of axial coordination for the guide of rational design and synthesis of axially coordinated SACs is also essential.

(2) Due to the intricate nature of the local coordination environment of SACs, especially those with high coordination numbers, the exact position of the axial ligand needs to be rigorously scrutinized. So far, reported axial coordination design of SACs is predominantly confirmed by synchrotron XAS study. However, it heavily relies on empirical interpretation and data quality. In addition, in certain cases it is hard to differentiate whether the ligand atom is located in the axial direction or the second coordination sphere. Therefore, meticulous study is necessary to prevent interference from the pseudo-coordination of surrounding atoms in proximity to the single atom sites. And the complementary synchrotron techniques like high-energy-resolution fluorescence-detected XANES (HERFD-XANES) and valence-to-core X-ray emission spectroscopy (V2C XES) are believed to provide a great help.

(3) Till now, the majority of axial ligands reported for SACs are restricted to nonmetallic heteroatoms as summarized in this review. Although very few studies on axial coordination by metal atoms has been reported, particularly the metal clusters, the related research is rarely explored. Since there have been already numerous reports on diatomic site catalysts, which generally exhibit higher activity than SACs due to the unique synergy of the adjacent metal atoms. Besides, there is also a rise in study of the concerted catalysis between SACs and metal clusters owing to the activity enhancement. Thereby, the cultivation on diverse axial coordination design of metal clusters to SACs holds great promise in acquiring much improved catalytic performance.

(4) The structural advantages of axial coordination modification of SACs have to be clarified. As shown in this review, axial coordination design is not always a panacea for the performance enhancement. In a few cases, it is not so catalytically active as the typical planar M–N_4_ coordination structure. Therefore, a systematic investigation into the structure–property correlation of axially coordinated SACs is necessary, with particular attention paid to parameters such as the types, numbers, sizes and other physicochemical properties of axial ligands. Moreover, the dynamic coordination structural evolution of axially coordinated SACs during the reaction process remains unexplored and largely overlooked, posing a significant concern in this field. As such, deep insights from the in situ/operando studies during the catalytic process are of significant importance.

(5) Although the feasibility of axial coordination design for SACs has been verified and widely investigated in various energy-conversion related electrochemical reactions, their applications have mostly been limited to ORR and CO_2_RR. Therefore, there is significant potential for further exploration of the concept of axial coordination design in SACs for other electrocatalytic reactions, such as hydrogen oxidation reaction (HOR), nitrogen oxide reduction reaction (NORR), C–C/C–N coupling reactions, and even extending to the domains of photocatalysis, thermocatalysis, Li–S batteries and Zn–I_2_ batteries, etc. Additionally, further consideration must be given to their industrial applications in fine chemical productions.
